# Increasing jojoba-like wax ester production in *Saccharomyces cerevisiae* by enhancing very long-chain, monounsaturated fatty acid synthesis

**DOI:** 10.1186/s12934-019-1098-9

**Published:** 2019-03-11

**Authors:** Leonie Wenning, Christer S. Ejsing, Florian David, Richard R. Sprenger, Jens Nielsen, Verena Siewers

**Affiliations:** 1 Department of Biology and Biological Engineering, Systems and Synthetic Biology, Chalmers University of Technology, Kemivägen 10, 412 96 Gothenburg, Sweden; 2 Novo Nordisk Foundation Center for Biosustainability, Chalmers University of Technology, Kemivägen 10, 412 96 Gothenburg, Sweden; 3 Department of Biochemistry and Molecular Biology, VILLUM Center for Bioanalytical Sciences, University of Southern Denmark, 5230 Odense, Denmark; 4 Biopetrolia AB, Kemivägen 10, 412 96 Gothenburg, Sweden; 5 Novo Nordisk Foundation Center for Biosustainability, Technical University of Denmark, Kemitorvet, 2800 Kgs. Lyngby, Denmark

**Keywords:** *Saccharomyces cerevisiae*, Very long-chain monounsaturated fatty acids, Very long-chain monounsaturated fatty alcohols, Jojoba-like wax esters

## Abstract

**Background:**

Fatty acids (FAs) with a chain length of more than 18 carbon atoms (> C18) are interesting for the production of specialty compounds derived from these FAs. These compounds include free FAs, like erucic acid (C22:1-Δ13), primary fatty alcohols (FOHs), like docosanol (C22:0-FOH), as well as jojoba-like wax esters (WEs) (C38-WE to C44-WE), which are esters of (very) long-chain FAs and (very) long-chain FOHs. In particular, FAs, FOHs and WEs are used in the production of chemicals, pharmaceuticals and cosmetic products. Jojoba seed oil is highly enriched in diunsaturated WEs with over 70 mol% being composed of C18:1–C24:1 monounsaturated FOH and monounsaturated FA moieties. In this study, we aim for the production of jojoba-like WEs in the yeast *Saccharomyces cerevisiae* by increasing the amount of very long-chain, monounsaturated FAs and simultaneously expressing enzymes required for WE synthesis.

**Results:**

We show that the combined expression of a plant-derived fatty acid elongase (FAE/KCS) from *Crambe abyssinica* (*Ca*KCS) together with the yeast intrinsic fatty acid desaturase (FAD) Ole1p leads to an increase in C20:1 and C22:1 FAs in *S. cerevisiae*. We also demonstrate that the best enzyme candidate for C24:1 FA production in *S. cerevisiae* is a FAE derived from *Lunaria annua* (*La*KCS). The combined overexpression of *Ca*KCS and Ole1p together with a fatty acyl reductase (FAR/FAldhR) from *Marinobacter aquaeolei* VT8 (*Ma*FAldhR) and a wax synthase (WS) from *Simmondsia chinensis* (*Sci*WS) in a *S. cerevisiae* strain, overexpressing a range of other enzymes involved in FA synthesis and elongation, leads to a yeast strain capable of producing high amounts of monounsaturated FOHs (up to C22:1-FOH) as well as diunsaturated WEs (up to C46:2-WE).

**Conclusions:**

Changing the FA profile of the yeast *S. cerevisiae* towards very long-chain monounsaturated FAs is possible by combined overexpression of endogenous and heterologous enzymes derived from various sources (e.g. a marine copepod or plants). This strategy was used to produce jojoba-like WEs in *S. cerevisiae* and can potentially be extended towards other commercially interesting products derived from very long-chain FAs.

**Electronic supplementary material:**

The online version of this article (10.1186/s12934-019-1098-9) contains supplementary material, which is available to authorized users.

## Background

Fatty acids (FAs) play a major role during growth and maintenance of all living cells, since they are components of cell membrane lipids, organelle membrane lipids as well as storage lipids. Moreover, they can function as signaling compounds in the form of sphingolipids. The FAs naturally found in the yeast *Saccharomyces cerevisiae* comprise mostly C16 and C18 species. The most abundant one is palmitoleic acid (C16:1-Δ9), followed by oleic acid (C18:1-Δ9), palmitic acid (C16:0) and stearic acid (C18:0) [1]. The composition of FAs in yeast is strictly regulated and dependent, among other factors, on the cultivation temperature [2] as well as the media composition. Culture medium that contains a certain type of FA can change the distribution of FAs inside the yeast cell [3].

The total distribution of FAs is also reflected by the composition of membrane- as well as storage lipids in yeast. In case of phospholipids (PLs), which make up around 70% of the cell membrane, the main FA residues are C14:1-Δ9, C16:1-Δ9 and C18:1-Δ9, which shows the importance of those FA species for the integrity of cell membranes in *S. cerevisiae*. In case of triacylglycerols (TAGs), which function as storage lipids, the most abundant FA residues are C16:0, C16:1-Δ9, C18:0 and C18:1-Δ9 [4]. Besides having a storage function in yeast, TAGs also act as a buffer and sink for excess unsaturated FAs [5] and reflect the composition of added or synthesized FAs [4]. The addition of oleic acid to the growth medium of yeast cells has a strong effect on lipid metabolism and the composition of storage lipids. While cells grown on glucose show approximately a 50/50 accumulation of the storage lipids steryl esters (SEs) and TAGs, cells grown on oleate show a ratio of 1/99. Moreover, the composition of PLs and TAGs changes towards an increased incorporation of oleic acid in medium supplemented with oleate [4]. Yeast cells unable of producing storage lipids, by deletion of genes encoding SE and TAG formation enzymes (Lro1p, Dga1p, Are1p and Are2p), show a defective regulation of lipid synthesis, an extensive proliferation of intracellular membranes and finally cell death under excess of oleate [5].

**Fig. 1:**
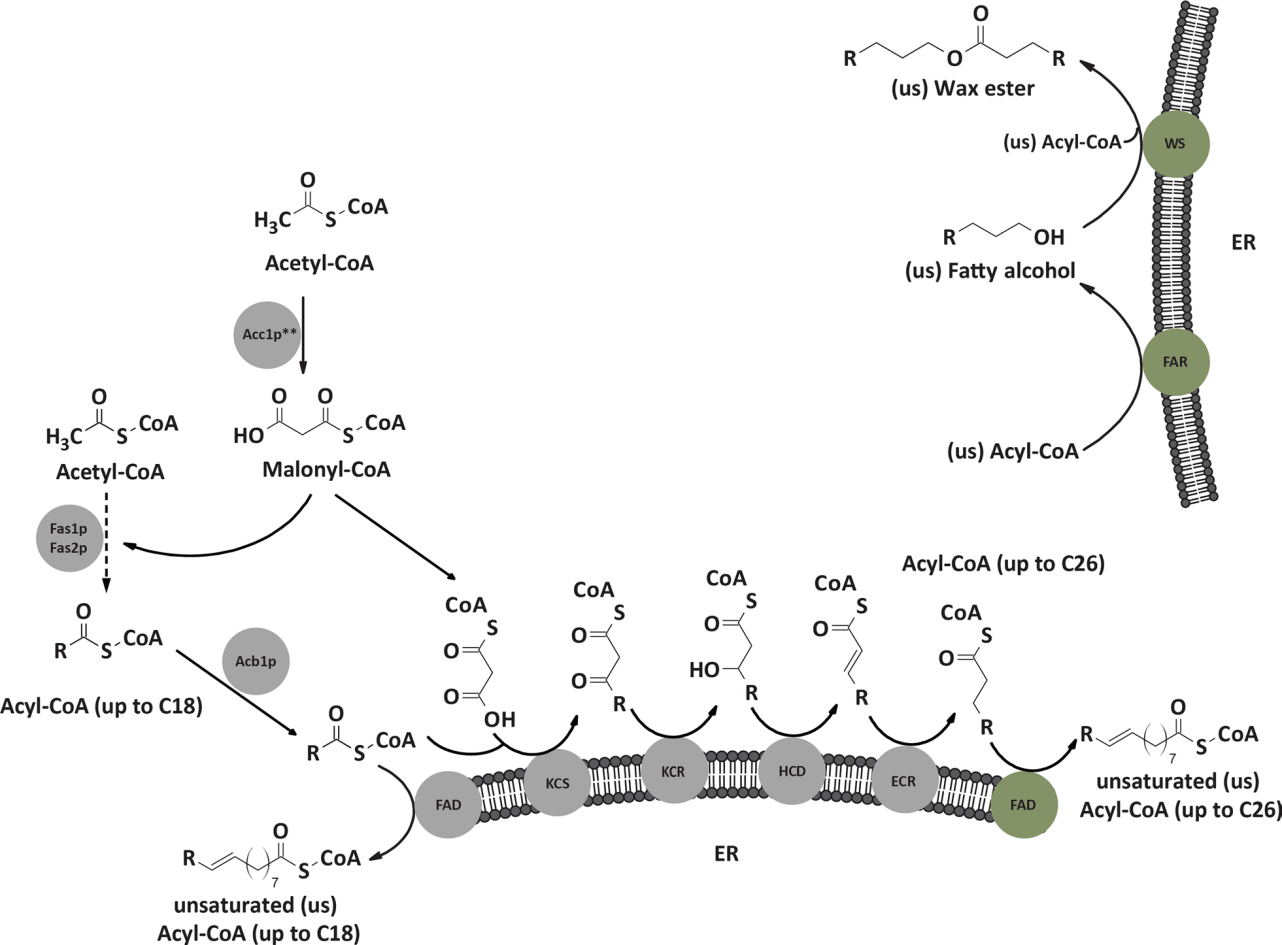
De novo synthesis and elongation of fatty acids (FAs), fatty alcohols (FOHs) and wax esters (WEs) in *S. cerevisiae*. The first step shown in the reaction scheme is the carboxylation of acetyl-CoA to malonyl-CoA catalyzed by acetyl-CoA carboxylase (Acc1p) in the cytosol. In our study, a mutant version of Acc1p, containing two amino acid substitutions (S659A; S1157A), was used (Acc1p**). The next step in the synthesis of FAs in the cytosol is a series of reactions catalyzed by the fatty acid synthases 1 (Fas1p) and 2 (Fas2p) which leads to fatty acyl CoAs (FACoAs) with a chain length of C12-C18. The acyl-CoA binding protein (Acb1p) transports newly synthesized acyl-CoA esters from Fas1p–Fas2p to acyl-CoA-consuming processes. The elongation and desaturation of C12-C18 FACoAs is performed at the endoplasmic reticulum (ER). The desaturation of FAs in *S. cerevisiae* is catalyzed by Ole1p, a fatty acid desaturase (FAD) acting on C12-C19 FAs. This scheme also shows a heterologous FAD (green) which is able to insert a double bond into very long-chain FAs (C20-C26). The elongation of FAs in *S. cerevisiae* is catalyzed by a β-ketoacyl-CoA synthase (KCS), a β-ketoacyl-CoA reductase (KCR), a β-hydroxyacyl-CoA dehydratase (HCD) and an enoyl-CoA reductase (ECR) and leads to the production of C16-C26 FACoAs in yeast. FACoAs can be reduced by a heterologous fatty acyl-CoA reductase (FAR) to a fatty aldehyde and further to a FOH. A wax synthase (WS) catalyzes the esterification of a FOH with another FACoA molecule to form a WE. All yeast intrinsic enzymes are indicated in grey, whereas heterologous ones are indicated in green

The de novo FA synthesis in *S. cerevisiae* is located in the cytosol and uses acetyl-coenzyme A (acetyl-CoA) as a building block. The first step of FA synthesis is the carboxylation of acetyl-CoA to malonyl-CoA by an acetyl-CoA carboxylase (Acc1p) [6]. Studies using a deregulated version of Acc1p, in which the serine 1157 was mutated to an alanine residue to lock the Acc1p in a non-phosphorylatable, highly active state, showed that the ratio of C18 to C16 FAs changed. In wildtype cells, the C18/C16 ratio was found to be around 0.5, while the deregulated Acc1p mutant strain showed a ratio of around 2 [7]. This mutation also led to an increase in very long-chain fatty acids (VLCFAs) (C20-C26). In the second step of FA synthesis, a C2 unit derived from malonyl-CoA is attached to acetyl–acyl carrier protein (acetyl-ACP) and in the further extensions to the acyl-ACP chain, leading to FAs up to C18. These reactions are in the cytosol catalyzed by the fatty acid synthase (FAS) consisting of Fas1p (β-subunit) and Fas2p (α-subunit), which form a hexameric α6β6 complex. When the FAs are released from the FAS enzyme complex, a coenzyme-A unit is attached to them [6]. Besides originating from de novo FA synthesis, external free FAs or free FAs derived from lipid or lipoprotein turnover can be activated to fatty acyl-CoAs (FACoAs) by FACoA synthetases [8–11]. In *S. cerevisiae*, FAs up to C18 can be further elongated up to C26 at the endoplasmic reticulum (ER). These reactions are catalyzed by the β-ketoacyl-CoA synthases (KCSs), also known as fatty acid elongases (FAEs), the β-ketoacyl-CoA reductase (KCR), the β-hydroxyacyl-CoA dehydratase (HCD) and the enoyl-CoA reductase (ECR) which are all integrated in the ER membrane and face the cytosol with their active site [6] (Fig. 1). *S. cerevisiae* possesses three KCS enzymes, Elo1p, Elo2p and Elo3p which all have a discrete substrate range. Elo1p elongates C12–C16 FACoAs to C16–C18 FACoAs. Elo2p is responsible for the biosynthesis of C20–C24 FACoAs and Elo3p specifically elongates C24 FACoAs to C26 FACoAs [12, 13]. In contrast to the KCS enzymes, which are substrate specific, the other elongation enzymes (KCR, HCD and ECR) show a broad specificity, accepting a wide range of substrates [6]. The elongation enzymes at the ER together fulfill the same function as the FAS complex in the cytosol, with the exception that the ER enzymes are distinct proteins in contrast to the FAS complex which contains two subunits with several functional domains.

Besides synthesis and elongation of FAs, also the desaturation of FAs plays an important role in the yeast cell metabolism. *S. cerevisiae* possesses only one fatty acid desaturase (FAD), Ole1p, which can integrate a double bond at the Δ9-position of FACoAs ranging from a carbon chain length of C12–C19 [14] (Fig. 1). Therefore, in contrast to other fungi, *S. cerevisiae* only contains monounsaturated fatty acids (MUFAs) with ~ 70–80% of its total glycerolipid acyl chains consisting of C14:1-Δ9, C16:1-Δ9 and C18:1-Δ9 [15].

Since the composition of FAs is very important for the integrity of membrane lipid structures, it can be expected that the yeast FA pool can only be changed to a certain extent. Nevertheless, a change of the FA pool can be interesting if certain compounds with a specific chain length (> C18) shall be produced. Those products include free fatty acids (FFAs), fatty alcohols (FOHs), alkanes/alkenes, lipids as well as speciality FAs like eicosapentaenoic acid and have been produced by metabolic engineering of *S. cerevisiae* and other yeasts [16].

Several commercially interesting products are also derived from very long-chain fatty acids (VLCFAs) and comprise very long-chain fatty alcohols (VLCFOHs) like docosanol (C22:0-FOH) [17] as well as jojoba-like wax esters (WEs), which are mainly composed of C18–C24 monounsaturated fatty alcohol (MUFOH) and monounsaturated fatty acid (MUFA) residues [18] (Fig. 1). The application of jojoba oil ranges from cosmetic and personal care products over medical use to lubricants. The current production of jojoba oil is around 4000 tons/year, while the estimated demand is up to 200,000 tons/year [19]. Because of the increased need for jojoba oil, extraction from its natural source will not be enough to meet the demand in the future, even if growing regions for the jojoba plant are expanded extensively. Since *S. cerevisiae* has already been modified for the production of a wide range of chemicals [20], jojoba oil production in modified yeast represents a very promising approach.

In a previous study we could show that jojoba-like WEs can be synthesized in metabolically engineered *S. cerevisiae* by heterologous expression of a FAR from *Marinobacter aquaeolei* VT8 and a WS from *Simmondsia chinensis*, but the products were restricted to saturated very long-chain WEs (C40:0-WE and C42:0-WE) [21]. This study made clear that the pool of very long-chain monounsaturated fatty acids (VLCMUFAs) needs to increase in *S. cerevisiae* to enable the synthesis of jojoba-like diunsaturated wax esters (DUWEs).

To achieve this goal, we here expressed several endogenous and heterologous KCSs and FADs in *S. cerevisiae* and demonstrate that this can lead to a high increase in C20:1, C22:1 and C24:1 MUFAs. Moreover, it is shown that the combined expression of a plant KCS, a bacterial FAR, a plant WS and several yeast intrinsic enzymes leads to a final MUFOH and DUWE producing strain.

## Results

### Increasing the amount of MUFAs by expression of a heterologous KCS together with Ole1p of *S. cerevisiae*

Previously, we demonstrated that jojoba-like WEs can be synthesized in metabolically engineered *S. cerevisiae* strains by combined overexpression of the yeast intrinsic Elo2p together with a FAR from *M. aquaeolei* and a WS from *S. chinensis*, but the products were restricted to saturated very long-chain WEs (C40:0-WE and C42:0-WE) [21]. Based on this observation we concluded that the pool of VLCMUFAs is not high enough to enable the synthesis of jojoba-like DUWEs in *S. cerevisiae*.

Therefore, we investigated the effect of overexpressing the endogenous Ole1p either together with the yeast intrinsic Elo2p or together with a heterologous plant-derived FAE/KCS enzyme in *S. cerevisiae.* The heterologous FAE/KCS enzymes tested include those from *Arabidopsis thaliana* (*At*FAE), *Brassica napus* (*Bn*KCS), *Crambe abyssinica* (*Ca*KCS)*, Lunaria annua* (*La*KCS), *S. chinensis* (*Sci*FAE) and *Tropaeolum majus* (*Tm*KCS). Combinations of a KCS and Ole1p were cloned into the episomal plasmid pYX212 via modular pathway engineering [22] (Additional file 1: Figure S1). The plasmids were transferred into the background strain *S. cerevisiae* CEN.PK 113-5D *elo3*Δ X-2::p*MPC3*::ACC1** X-3::*IFA38*::*PHS1*::*TSC13*::*ACB1*. This strain contains a constitutively active version of Acc1p to increase the supply of malonyl-CoA, a deletion of the *ELO3* gene to prevent formation of C26 FAs and an additional copy of the genes *IFA38*, *PHS1* and *TSC13*, encoding the yeast intrinsic elongation enzymes KCR, HCD and ECR, respectively. Moreover, it harbors an additional copy of the *ACB1* gene encoding the acyl-CoA binding protein Acb1p to increase the acyl-CoA pool, which has been shown in a previous study to result in an increased fatty acid ethyl ester production [23].

**Fig. 2:**
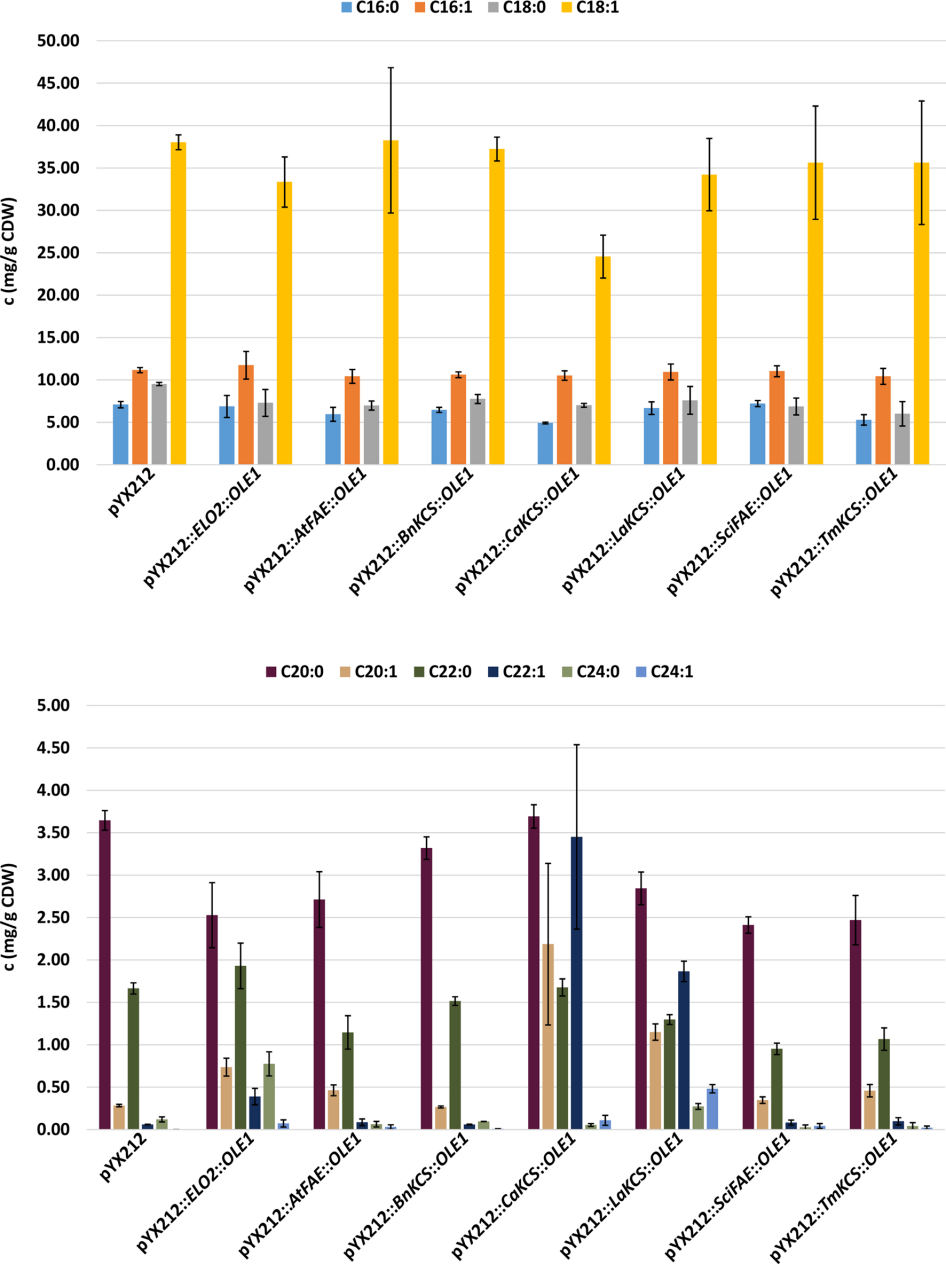
Effect of expression of (heterologous) elongases (FAEs/KCSs) together with the yeast intrinsic Ole1p desaturase on the concentration of fatty acids (FAs) (mg/g CDW) in the background strain CEN.PK 113-5D *elo3*Δ X-2::p*MPC3*::ACC1** X-3::*IFA38*::*PHS1*::*TSC13*::*ACB1* (LW03). The values represent the mean ± standard deviation (SD) of three biological replicates of strains LW04 (pYX212), LW05 (pYX212::*ELO2*::*OLE1*), LW06 (pYX212::*AtFAE*::*OLE1*), LW07 (pYX212::*BnKCS*::*OLE1*), LW08 (pYX212::*CaKCS*::*OLE1*), LW09 (pYX212::*LaKCS*::*OLE1*), LW10 (pYX212::*SciFAE*::*OLE1*) and LW11 (pYX212::*TmKCS*::*OLE1*), respectively. The strains were grown for 48 h in minimal medium containing 20 g/L glucose

The analysis of the FA profile of the resulting strains showed that the combined overexpression of a KCS together with the yeast intrinsic Ole1p leads to an increase in the pool of VLCMUFAs in case of the KCS enzymes Elo2p, *Ca*KCS and *La*KCS. The KCS enzymes from *A. thaliana*, *B. napus* and *T. majus* were not able to increase the pool of VLCMUFAs (Fig. 2).

The highest increase in C20:1 and C22:1 FAs in our study was obtained by combined expression of the KCS from *C. abyssinica* (*Ca*KCS) together with the intrinsic Ole1p from *S. cerevisiae*. The level of C20:1 FAs and C22:1 FAs increased 20.6- and 95.7-fold, respectively, compared to the wildtype. In contrast to that, the highest increase in C24:1 FAs (25.6-fold compared to Elo2p overexpression strain) was observed by expression of the KCS from *L. annua* (*La*KCS) together with Ole1p (Fig. 2).

### Increasing the amount of MUFAs by expression of a heterologous KCS and a heterologous FAD

After having identified *Ca*KCS and *La*KCS as promising enzyme candidates to increase the C20:1, C22:1 and C24:1 FA concentrations in *S. cerevisiae*, we wanted to study the influence of heterologous FADs on the synthesis of VLCMUFAs. The heterologous FAD enzymes tested include those from *C. hyperboreus* (*Ch*Des9-1) and *S. chinensis* (*Sci*FAD-SP) as well as two acyl- CoA desaturase-like (ADS) proteins from *A. thaliana* (*At*ADS1.2 and *At*ADS1.4). Combinations of *Ca*KCS or *La*KCS and a heterologous FAD were cloned into the episomal plasmid pYX212 via modular pathway engineering [22] (Additional file 1: Figure S1). The plasmids were transferred into the background strain *S. cerevisiae* CEN.PK 113-5D *elo3*Δ X-2:pMPC3::ACC1** X-3::*IFA38*::*PHS1*::*TSC13*::*ACB1*.

**Fig. 3:**
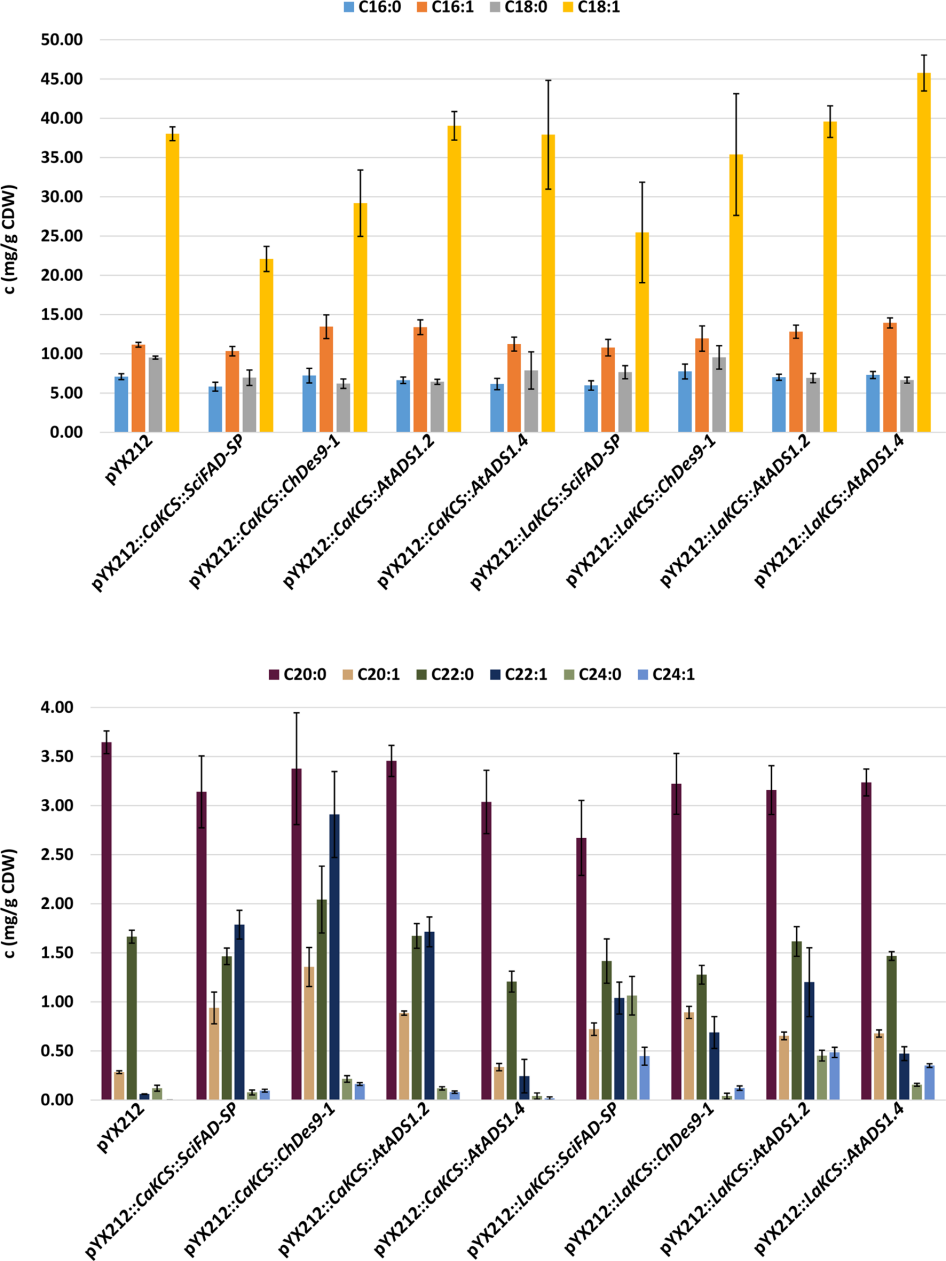
Effect of expression of (heterologous) elongases (FAEs/KCSs) together with heterologous desaturases (FADs) on the concentration of fatty acids (FAs) (mg/g CDW) in the background strain CEN.PK 113-5D *elo3*Δ X-2::p*MPC3*::ACC1** X-3::*IFA38*::*PHS1*::*TSC13*::*ACB1* (LW03). The values represent the mean ± standard deviation (SD) of three biological replicates of strains LW04 (pYX212), LW12 (pYX212::*CaKCS*::*SciFAD*-*SP*), LW13 (pYX212::*CaKCS*::*ChDes9*-*1*), LW14 (pYX212::*CaKCS*::*AtADS1.2*), LW15 (pYX212::*CaKCS*::*AtADS1.4*), LW16 (pYX212::*LaKCS*::*SciFAD*-*SP*), LW17 (pYX212::*LaKCS*::*ChDes9*-*1*), LW18 (pYX212::*LaKCS*::*AtADS1.2*) and LW19 (pYX212::*LaKCS*::*AtADS1.4*), respectively. The strains were grown for 48 h in minimal medium containing 20 g/L glucose

The highest amount of C20:1 and C22:1 FAs (1.36 ± 0.20 mg FAs/g CDW and 2.91 ± 0.44 mg FAs/g CDW, respectively), accompanied by a decrease in C18:1 FAs of 23.3% (compared to strain LW04), was observed in a strain expressing *Ca*KCS together with *Ch*Des9-1. This is slightly less compared with the amount of VLCMUFAs produced in the strain expressing *Ca*KCS together with Ole1p. The production of C24:1 FAs was highest in a strain expressing *La*KCS together with an ADS protein from *A. thaliana* (*At*ADS1.2) (Fig. 3).

Therefore, after evaluation of the combined expression of *Ca*KCS or *La*KCS with various FADs, the combination of *Ca*KCS and Ole1p was identified as best enzymes for high level production of C20:1 and C22:1 FAs and chosen to be expressed together with the WE biosynthesis enzymes *Ma*FAldhR and *Sci*WS to further boost WE production.

### Integration of pathway genes to construct MUFOH and DUWE production strain

The overexpression cassettes for *Ca*KCS, Elo1p, Faa1p and Ole1p were integrated into the genome of *S. cerevisiae*. The combined overexpression of *Ca*KCS, Elo1p, Faa1p and Ole1p (strain LW22) led to a growth defect of the strain with a final OD_600_ of 2.62 ± 0.05 after 48 h, compared with the same strain lacking the overexpression of *Ca*KCS and Ole1p (strain LW21) which showed a final OD_600_ of 10.9 ± 0.18 after 48 h. This growth defect was also observed in strain LW24, expressing *Ca*KCS and Ole1p in combination with the WE synthesizing enzymes *Ma*FAldhR and *Sci*WS as well as Elo2p, but not in strain LW23, expressing *Ma*FAldhR, *Sci*WS and Elo2p, but lacking *Ca*KCS and Ole1p (Additional file 1: Figure S3).

The integration-based expression of *Ca*KCS, Elo1p, Faa1p and Ole1p together with the plasmid-based expression of *Ma*FAldhR, Elo2p and *Sci*WS led to appearance of two additional bands on the thin layer chromatography (TLC) plate in strain LW23 (expressing only Elo1p, Elo2p, Faa1p*, Ma*FAldhR and *Sci*WS) as well as in strain LW24 (expressing *Ca*KCS, Elo1p, Elo2p, Faa1p, *Ma*FAldhR, Ole1p and *Sci*WS) (Additional file 1: Figures S4, S5).

The analysis via gas chromatography-flame ionization detector (GC-FID) and gas chromatography-mass spectrometry (GC/MS) showed that those additional bands on the TLC plate corresponded to FOHs and WEs, respectively (Additional file 1: Figures S6, S7). The control strains LW21 and LW22, which do not contain the plasmid pYX212::*MaFAldhR*::*SciWS*::*ELO2,* did not show peaks corresponding to FOHs or WEs. In contrast to that, strain LW23 produced C16:0-FOH, C18:0-FOH, C18:1-FOH, C20:0-FOH and C22:0-FOH (Additional file 1: Figure S6). These FOH species could also be detected in strain LW24, which additionally produced C16:1-FOH, C20:1-FOH and C22:1-FOH (Additional file 1: Figure S6). The analysis of WEs via GC/MS showed that both LW23 and LW24 strains produced WEs in the chain length range of C30-WE to C44-WE (Additional file 1: Figure S7), but only in case of strain LW24, DUWE synthesis could be detected. The synthesis of C38:2-WE, C40:2-WE, C42:2-WE and C44:2-WE was confirmed by analysis of the mass spectra of the corresponding GC peaks, which showed the appearance of m/z peaks corresponding to the molecular ions of these DUWEs (m/z = 561, 589, 617 and 645, respectively) (Additional file 1: Figure S8). Those DUWE specific m/z peaks could not be detected in the strain lacking the expression of *Ca*KCS and Ole1p (strain LW23).

**Fig. 4:**
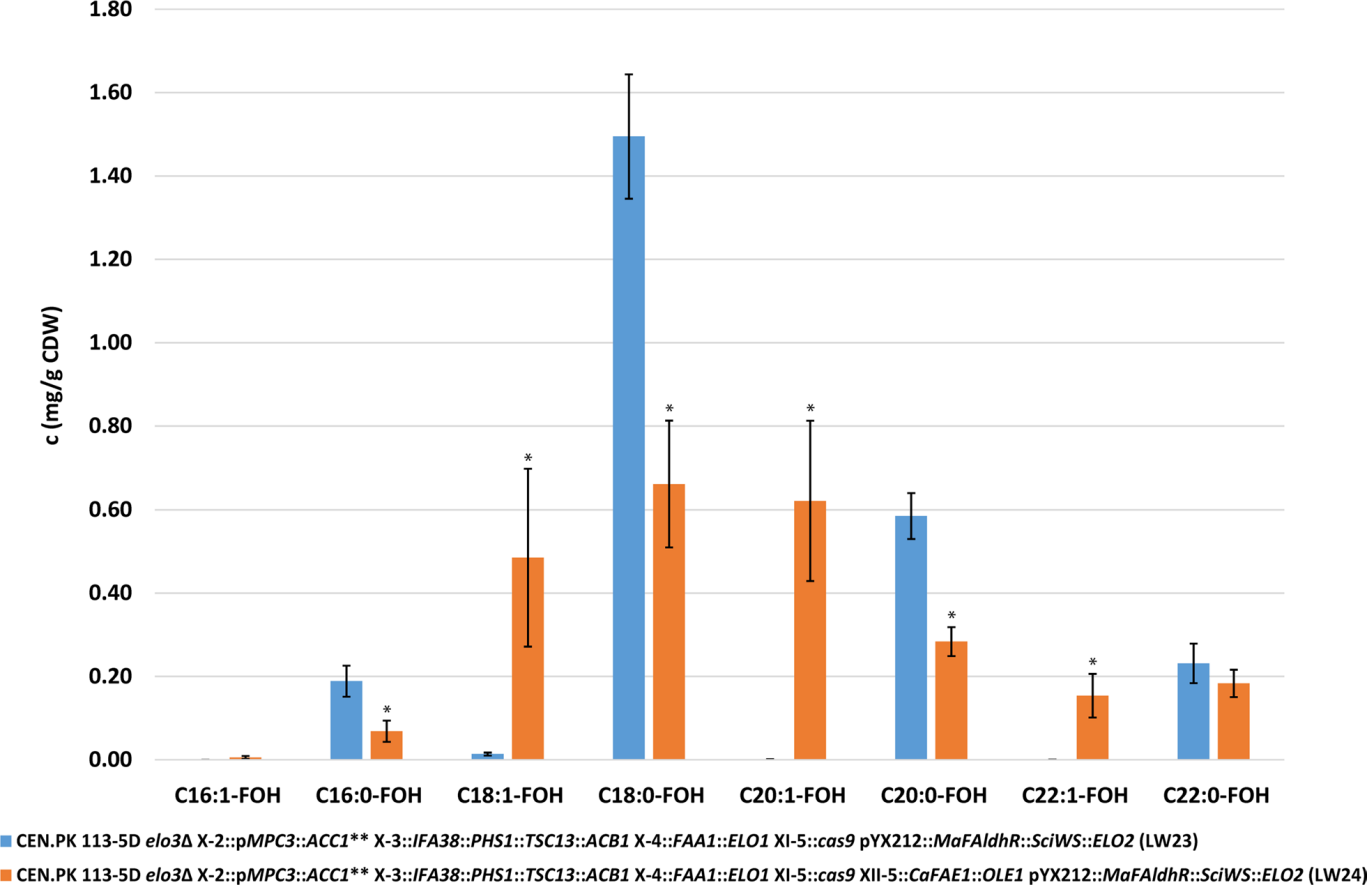
Concentration of fatty alcohols (FOHs) (mg/g CDW). The values represent the mean ± standard deviation (SD) of three biological replicates of strains LW23 (expressing only Elo1p, Elo2p, Faa1p*, Ma*FAldhR and *Sci*WS) and LW24 (expressing *Ca*KCS, Elo1p, Elo2p, Faa1p, *Ma*FAldhR, Ole1p and *Sci*WS), respectively. The strains were grown for 48 h in minimal medium containing 20 g/L glucose. *p < 0.05 (Students t-test, two-tailed, unequal variance assumed)

The quantification of FOHs and WEs showed that the expression of *Ca*KCS and Ole1p in strain LW24 leads to the production of 1.27 ± 0.46 mg MUFOHs/g CDW, with a significant increase (p < 0.05) in the MUFOHs C18:1-FOH, C20:1-FOH and C22:1-FOH and a significant decrease (p < 0.05) in the saturated FOHs C16:0-FOH, C18:0-FOH and C20:0-FOH compared to strain LW23. In total, strain LW24 produced 2.46 ± 0.71 mg FOHs/g CDW. In contrast to that, the strain lacking the expression of *Ca*KCS and Ole1p (strain LW23) produced 2.51 ± 0.29 mg FOHs/g CDW, of which 0.014 mg ± 0.005/g CDW were MUFOHs. This corresponds to an increase in MUFOHs by 89.5-fold in strain LW24 compared to strain LW23 (Fig. 4). The overall titer of FOHs was 6.92 ± 0.85 mg/L in strain LW23 and 2.10 ± 0.55 mg/L in strain LW24, which corresponds to a yield of 0.346 mg/g glucose and 0.105 mg/g glucose, respectively.

**Fig. 5:**
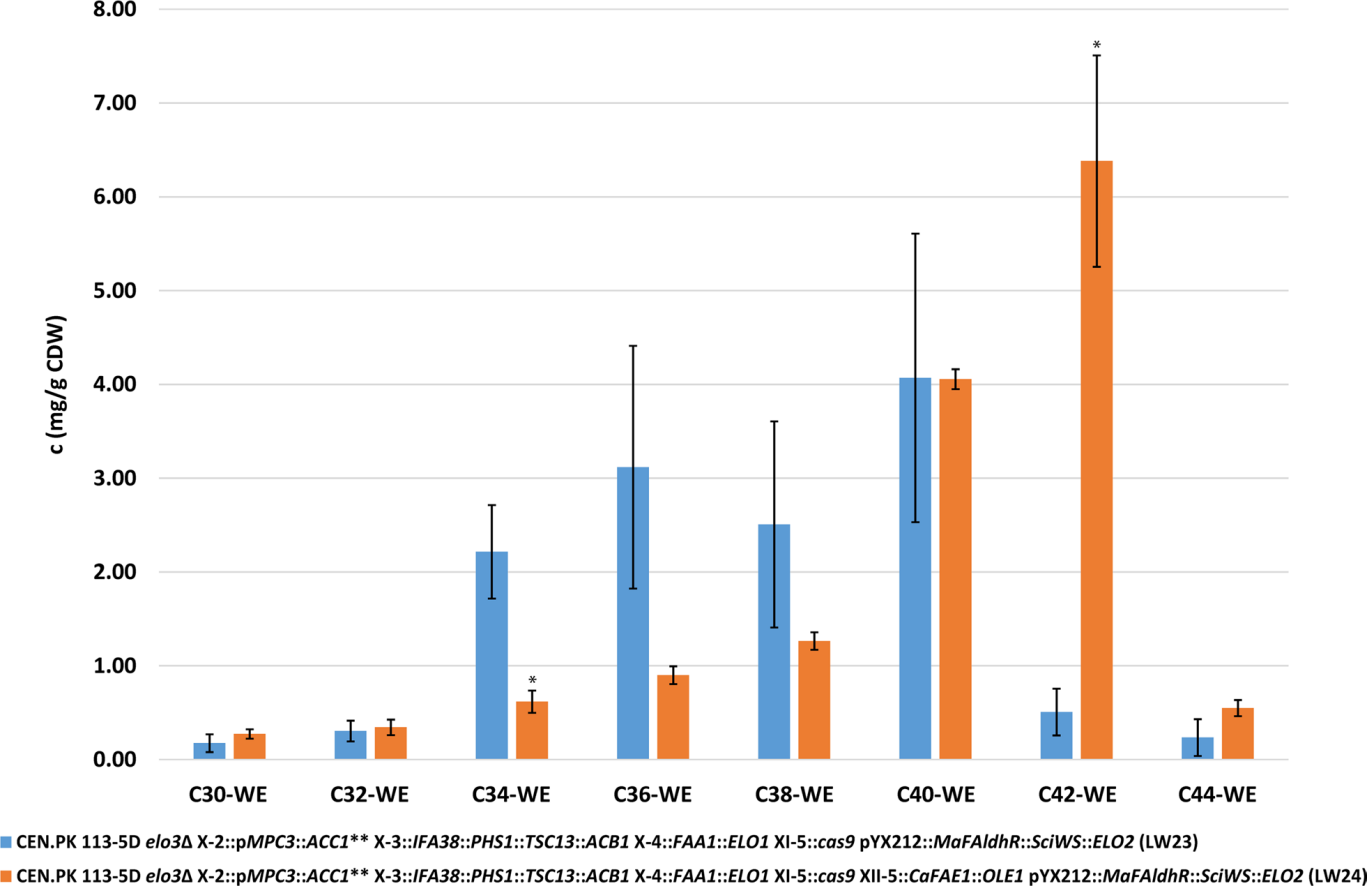
Concentration of wax esters (WEs) (mg/g CDW). The values represent the mean ± standard deviation (SD) of three biological replicates of strains LW23 (expressing only Elo1p, Elo2p, Faa1p*, Ma*FAldhR and *Sci*WS) and LW24 (expressing *Ca*KCS, Elo1p, Elo2p, Faa1p, *Ma*FAldhR, Ole1p and *Sci*WS), respectively. The strains were grown for 48 h in minimal medium containing 20 g/L glucose. *p < 0.05 (Students t-test, two-tailed, unequal variance assumed)

**Fig. 6:**
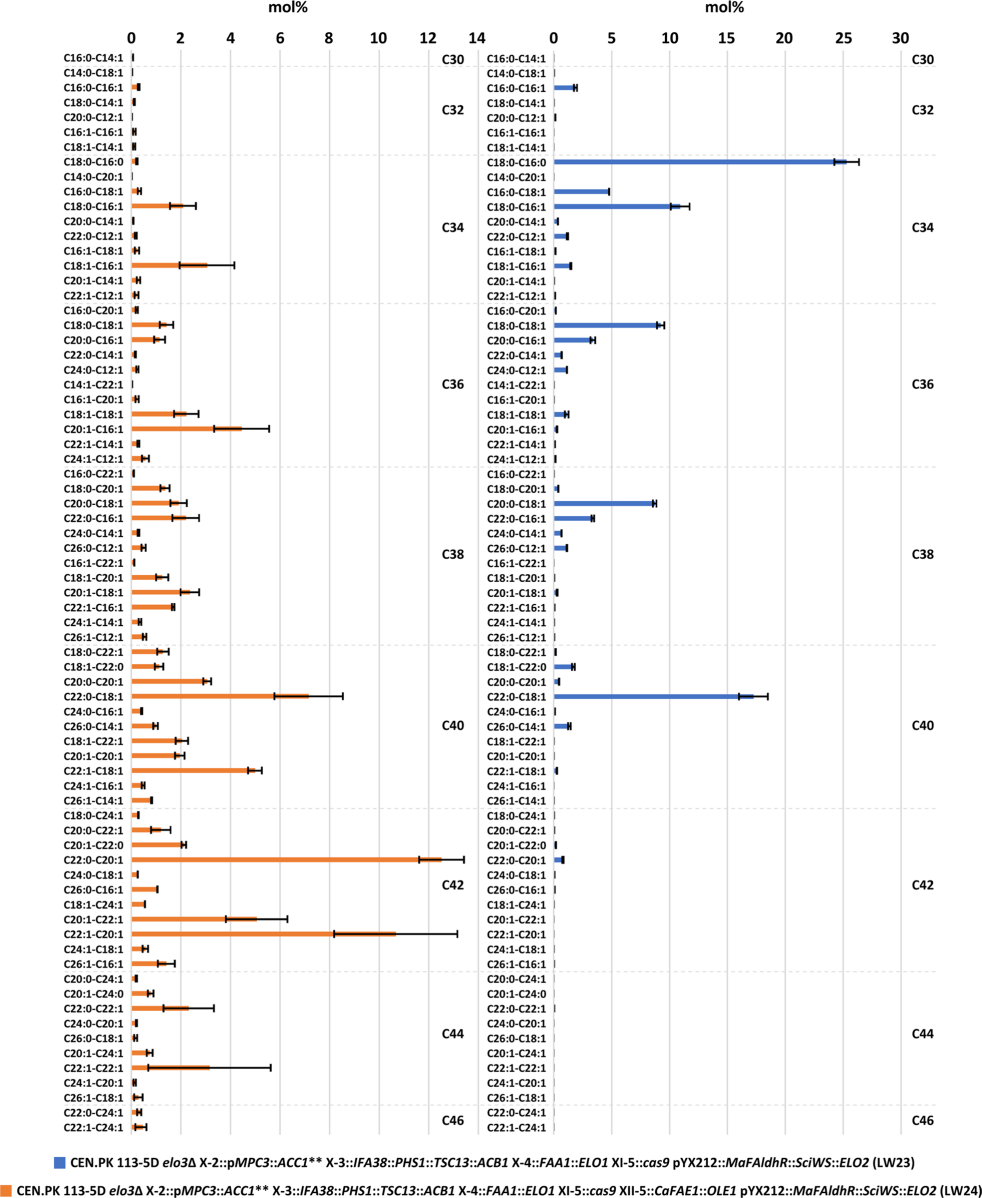
Molecular composition of wax ester (WE) species (mol%). The values represent the mean ± standard deviation (SD) of three biological replicates of strains LW23 (expressing only Elo1p, Elo2p, Faa1p*, Ma*FAldhR and *Sci*WS) and LW24 (expressing *Ca*KCS, Elo1p, Elo2p, Faa1p, *Ma*FAldhR, Ole1p and *Sci*WS), respectively. The strains were grown for 48 h in minimal medium containing 20 g/L glucose

This increase in MUFOHs was accompanied by the formation of DUWEs in strain LW24. The quantification of WEs in strains LW23 and LW24 showed that in total 13.13 ± 5.08 mg WEs/g CDW and 14.38 ± 1.76 mg WEs/g CDW, respectively, were produced. Strain LW24 showed a significant decrease (p < 0.05) in C34-WE species and a significant increase (p < 0.05) in C42-WE species (Fig. 5). The overall titer of WEs was 36.5 ± 16.71 mg/L in strain LW23 and 11.92 ± 1.47 mg/L in strain LW24, which corresponds to a yield of 1.83 mg/g glucose and 0.60 mg/g glucose, respectively. The C40-WE to C44-WE made up 36.6% in strain LW23, while in strain LW24, 76.4% of the WEs were C40-WE to C44-WE. The analysis of the molecular composition of WEs in strains LW23 and LW24 led to an overall identification of 73 WE species (Fig. 6). The most abundant WE species in strain LW23 were C18:0-C16:0 (25.30 ± 1.07 mol%), followed by C22:0–C18:1 (17.25 ± 1.24 mol%) and C18:0–C16:1 (10.92 ± 0.82 mol%). In contrast to that, strain LW24 produced WEs that contain longer and more unsaturated FOH and FA moieties, with the most abundant WE species being C22:0–C20:1 (12.52 ± 0.90 mol%), C22:1–C20:1 (10.67 ± 2.48 mol%) and C22:0–C18:1 (7.16 ± 1.38 mol%). Additionally, low amounts of C46-WE could be identified in strain LW24 (Additional file 1: Table S3). This represents the first study in which the production of DUWEs of a chain length of C38-WE to C46-WE was achieved in the yeast *S. cerevisiae*.

## Discussion

In this study we aimed for the increased production of VLCMUFAs in the yeast *S. cerevisiae* as precursors for the synthesis of jojoba-like DUWEs. For this purpose, we have screened a range of endogenous and heterologous FAEs/KCSs and FADs. It could be shown that the KCSs derived from the plants *C. abyssinica* (*Ca*KCS) and *L. annua* (*La*KCS) as well as the yeast intrinsic Elo2p are able to increase the pool of VLCMUFAs when expressed together with the yeast intrinsic FAD Ole1p. In contrast to that, the KCS enzymes from *A. thaliana*, *B. napus, S. chinensis* as well as *T. majus* failed to increase the concentration of FAs (Fig. 2). *A. thaliana* and *B. napus* enzymes have been expressed previously in *S. cerevisiae* and were shown to be able to increase the pool of VLCFAs [24, 25]. However, in those studies no codon optimized sequences were used, and the enzymes were expressed in a different *S. cerevisiae* background strain (INVSc1), which might explain the different results observed in our study.

The first isolation and functional characterization of *Ca*KCS has been performed by Mietkiewska et al. [26]. They heterologously expressed *Ca*KCS in *S. cerevisiae* strain INVSc1 and showed that the strain produces the non-native to yeast FAs C20:1-Δ11, C20:1-Δ13, C22:1-Δ13, C22:1-∆15, C26:1-Δ17 and 26:1-∆19. Enzyme assays demonstrated that the preferred substrate of the enzyme is C20:1-CoA, and erucic acid (C22:1-Δ13) was synthesized as main product [26]. The heterologous expression of *La*KCS in the *S. cerevisiae* strain INVSc1 led to the synthesis of C20:1-Δ11, C22:0, C22:1-Δ13, C24:0, C24:1-Δ15 and C26:1-Δ17 FAs [27].

A recent study in the oleaginous yeast *Rhodosporidium toruloides*, in which the influence of the expression of several plant derived KCS enzymes on the VLCFA pool was investigated, also showed that the highest amount of C22:1 FAs (24% of total fatty acids (TFAs)) was observed under expression of the KCS derived from *C. abyssinica*. This increase in C22:1 FAs was accompanied by a 50% decrease in C18:1 FAs. In our *S. cerevisiae* strain, the expression of *Ca*KCS led to an erucic acid (C22:1-Δ13) content of 6% of TFAs and a 35.4% decrease in C18:1 FAs. In contrast to our study which identified the KCS from *L. annua* as best candidate for nervonic acid production, expression of *La*KCS in *R. toruloides* did not lead to a high increase in nervonic acid, but a high increase in eicosenoic acid (21.4% of TFAs). In *R. toruloides*, the highest production of nervonic acid (10% of TFAs) was obtained by expression of the KCS from *Cardamine graeca* [28]. This comparison shows that the expression of plant derived KCS enzymes can lead to different results, depending on the yeast species.

The heterologous FADs we tested to increase the amount of MUFAs in *S. cerevisiae* included those from *C. hyperboreus* (*Ch*Des9-1) and *S. chinensis* (*Sci*FAD-SP) as well as two ADS proteins from *A. thaliana* (*At*ADS1.2 and *At*ADS1.4). Our results indicate that the combined expression of *Ca*KCS and *Ch*Des9-1 can increase the amount of C20:1 and C22:1 FAs, but not as strong as the combined expression of *Ca*KCS and Ole1p. The production of C24:1 FAs was highest in a strain expressing *La*KCS together with *At*ADS1.2 (Fig. 3).

*Ch*Des9-1 was identified previously as a new type of Δ9-desaturase, which introduces a Δ9-double bond into VLCFAs ranging from C20:0 to C26:0 in its natural host, the marine copepod *C. hyperboreus*. When *Ch*Des9-1 was expressed in the *S. cerevisiae* strain INVSc1, the yeast mainly produced two new FAs, C20:1-Δ9 and C26:1-Δ9. Upon supplementation of the medium with C18:0, also C22:1-Δ9, C22:1-Δ11, C24:1-Δ9, C24:1-Δ11 and C24:1-Δ13 could be detected, besides the production of C20:1-Δ9 and C26:1-Δ9. The highest conversion efficiency was observed for C20:0 [29].

*At*ADS1.2 was characterized recently in an attempt to determine the biological function of seven members of the ADS gene family in *A. thaliana*, which in total contains nine genes encoding FAD-like proteins. The enzymes were expressed in the yeast strain *fat1*Δ (BY4741 *MAT*a *his3D1 leu2D0 met15D0 ura3D0 YBR041w::kanMX4*) from Invitrogen. Yeast cells expressing *At*ADS1.2 showed the synthesis of C22:1-Δ9, C24:1-Δ9 and C26:1-Δ9 FAs [30].

In contrast to the VLCMUFAs produced by *Ch*Des9-1 and *At*ADS1.2, which mostly contain the double bond in the Δ9-position, jojoba oil consists of C20:1-Δ11, C22:1-Δ13 and C24:1-Δ15 FAs, which result from the elongation of C18:1-Δ9 [31, 32]. This means that the VLCMUFAs produced in a yeast strain heterologously expressing *Ch*Des9-1 or *At*ADS1.2 probably do not represent the FA spectrum in jojoba oil. However, Ole1p introduces a double bond at the Δ9-position of FAs with a chain length up to C19 [14], indicating that a combined expression of a heterologous plant FAE/KCS together with the yeast intrinsic Ole1p can lead to the formation of jojoba-like VLCMUFAs in yeast by elongation of C18:1-Δ9 produced by Ole1p.

Therefore, the combination of *Ca*KCS and Ole1p was identified as best enzymes for high level production of C20:1 and C22:1 FAs in *S. cerevisiae* and chosen to be expressed together with the WE biosynthesis enzymes *Ma*FAldhR and *Sci*WS to further boost WE production. The *S. cerevisiae* strain expressing *Ca*KCS, Elo1p, Faa1p and Ole1p (strain LW22) showed a growth defect which was not detected in the same strain lacking the overexpression of *Ca*KCS and Ole1p (strain LW21). This growth defect was also observed in strain LW24, expressing *Ca*KCS and Ole1p in combination with the WE synthesizing enzymes *Ma*FAldhR and *Sci*WS as well as Elo2p, but not in strain LW23, expressing *Ma*FAldhR, *Sci*WS and Elo2p, but lacking *Ca*KCS and Ole1p (Additional file 1: Figure S3). This observation indicates that the growth defect is due to an increased synthesis of VLCMUFAs in strains LW22 and LW24 which seem to be toxic to the cells as has been shown before for C16:1-Δ9 and C18:1-Δ9 FAs [5].

The synthesis of VLCMUFAs in a strain expressing a FAR from *M. aquaeolei* (*Ma*FAldhR) and a WS from *S. chinensis* (*Sci*WS) (strain LW24) triggered the synthesis of MUFOHs (C16:1-FOH, C18:1-FOH, C20:1-FOH and C22:1-FOH) and DUWEs (up to C44:2-WE) (Figs. 4, 5, Additional file 1: Figures S6, S7, S8). The analysis of the molecular composition of the WEs led to the identification of 73 WE species (Fig. 6). Whereas strain LW23 mostly showed the production of saturated C34-WE (25.30 ± 1.07 mol%), strain LW24 showed a clear trend towards longer and more unsaturated WEs with the most abundant one being C42:2-WE (18.26 ± 4.18 mol%), followed by C42:1-WE (17.43 ± 0.63 mol%), C40:1-WE (14.01 ± 1.55 mol%) and C40:2-WE (10.29 ± 0.41 mol%) (Additional file 1: Table S3). This composition of WEs is similar to the one of jojoba oil, which consists mostly of C42:2-WE (46.8 mol%), followed by C40:2-WE (20.7 mol%), C44:2-WE (6.0 mol%) and C38:2-WE (4.3 mol%), with the exception that jojoba oil only contains DUWEs [18]. These results clearly demonstrate that the production of jojoba-like WEs is possible in *S. cerevisiae* under supply of appropriate precursors and that *Ma*FAldhR and *Sci*WS are enzymes with a broad substrate specificity.

## Conclusions

Our study shows that it is possible to drastically increase the amount of VLCMUFAs in *S. cerevisiae* by heterologous expression of a plant derived KCS together with a yeast intrinsic FAD. Moreover, we were able to show that the increase of VLCMUFAs can also trigger the synthesis of MUFOHs and DUWEs in a strain expressing a FAR and a WS. The general amount of jojoba-like WEs with a chain length of C40-WE to C44-WE was increased 2.3-fold by expression of a plant derived KCS together with a yeast intrinsic FAD compared to the same strain lacking the overexpression of those enzymes.

## Methods

### Strains and reagents

In this study, the strain *S. cerevisiae* CEN.PK 113-5D (*MAT*a *MAL2*-*8c SUC2 ura3*-*52*), kindly provided by P. Kötter (University of Frankfurt, Germany), was used as the parental strain. Yeast strains constructed based on this strain are listed in Table 1. For construction and maintenance of plasmids, *Escherichia coli* DH5α was used.

**Table 1:** List of strains constructed and used in this study

Name	Background	Plasmid/integration	Resource
CEN.PK 113-5D (LW00)	*MAT*a *MAL2*-*8c SUC2 ura3*-*52*	–	P. Kötter (University of Frankfurt, Germany)
LW01	*MAT*a *MAL2*-*8c SUC2 ura3*-*52 elo3*Δ	–	Wenning et al. [21]
LW02	*MAT*a *MAL2*-*8c SUC2 ura3*-*52 elo3*Δ X-2::p*MPC3*::ACC1**	–	This study
LW03	*MAT*a *MAL2*-*8c SUC2 ura3*-*52 elo3*Δ X-2::p*MPC3*::ACC1** X-3::*IFA38*::*PHS1*::*TSC13*::*ACB1*	–	This study
LW04	*MAT*a *MAL2*-*8c SUC2 ura3*-*52 elo3*Δ X-2::p*MPC3*::ACC1** X-3::*IFA38*::*PHS1*::*TSC13*::*ACB1*	pYX212	This study
LW05	*MAT*a *MAL2*-*8c SUC2 ura3*-*52 elo3*Δ X-2::p*MPC3*::ACC1** X-3::*IFA38*::*PHS1*::*TSC13*::*ACB1*	pYX212::*ELO2::OLE1*	This study
LW06	*MAT*a *MAL2*-*8c SUC2 ura3*-*52 elo3*Δ X-2::p*MPC3*::ACC1** X-3::*IFA38*::*PHS1*::*TSC13*::*ACB1*	pYX212::*AtFAE::OLE1*	This study
LW07	*MAT*a *MAL2*-*8c SUC2 ura3*-*52 elo3*Δ X-2::p*MPC3*::ACC1** X-3::*IFA38*::*PHS1*::*TSC13*::*ACB1*	pYX212::*BnKCS::OLE1*	This study
LW08	*MAT*a *MAL2*-*8c SUC2 ura3*-*52 elo3*Δ X-2::p*MPC3*::ACC1** X-3::*IFA38*::*PHS1*::*TSC13*::*ACB1*	pYX212::*CaKCS::OLE1*	This study
LW09	*MAT*a *MAL2*-*8c SUC2 ura3*-*52 elo3*Δ X-2::p*MPC3*::ACC1** X-3::*IFA38*::*PHS1*::*TSC13*::*ACB1*	pYX212::*LaKCS::OLE1*	This study
LW10	*MAT*a *MAL2*-*8c SUC2 ura3*-*52 elo3*Δ X-2::p*MPC3*::ACC1** X-3::*IFA38*::*PHS1*::*TSC13*::*ACB1*	pYX212::*SciFAE::OLE1*	This study
LW11	*MAT*a *MAL2*-*8c SUC2 ura3*-*52 elo3*Δ X-2::p*MPC3*::ACC1** X-3::*IFA38*::*PHS1*::*TSC13*::*ACB1*	pYX212::*TmKCS::OLE1*	This study
LW12	*MAT*a *MAL2*-*8c SUC2 ura3*-*52 elo3*Δ X-2::p*MPC3*::ACC1** X-3::*IFA38*::*PHS1*::*TSC13*::*ACB1*	pYX212::*CaKCS::SciFAD*-*SP*	This study
LW13	*MAT*a *MAL2*-*8c SUC2 ura3*-*52 elo3*Δ X-2::p*MPC3*::ACC1** X-3::*IFA38*::*PHS1*::*TSC13*::*ACB1*	pYX212::*CaKCS::ChDes9*-*1*	This study
LW14	*MAT*a *MAL2*-*8c SUC2 ura3*-*52 elo3*Δ X-2::p*MPC3*::ACC1** X-3::*IFA38*::*PHS1*::*TSC13*::*ACB1*	pYX212::*CaKCS::AtADS1.2*	This study
LW15	*MAT*a *MAL2*-*8c SUC2 ura3*-*52 elo3*Δ X-2::p*MPC3*::ACC1** X-3::*IFA38*::*PHS1*::*TSC13*::*ACB1*	pYX212::*CaKCS::AtADS1.4*	This study
LW16	*MAT*a *MAL2*-*8c SUC2 ura3*-*52 elo3*Δ X-2::p*MPC3*::ACC1** X-3::*IFA38*::*PHS1*::*TSC13*::*ACB1*	pYX212::*LaKCS::SciFAD*-*SP*	This study
LW17	*MAT*a *MAL2*-*8c SUC2 ura3*-*52 elo3*Δ X-2::p*MPC3*::ACC1** X-3::*IFA38*::*PHS1*::*TSC13*::*ACB1*	pYX212::*LaKCS::ChDes9*-*1*	This study
LW18	*MAT*a *MAL2*-*8c SUC2 ura3*-*52 elo3*Δ X-2::p*MPC3*::ACC1** X-3::*IFA38*::*PHS1*::*TSC13*::*ACB1*	pYX212::*LaKCS::AtADS1.2*	This study
LW19	*MAT*a *MAL2*-*8c SUC2 ura3*-*52 elo3*Δ X-2::p*MPC3*::ACC1** X-3::*IFA38*::*PHS1*::*TSC13*::*ACB1*	pYX212::*LaKCS::AtADS1.4*	This study
LW20	*MAT*a *MAL2*-*8c SUC2 ura3*-*52 elo3*Δ X-2::p*MPC3*::ACC1** X-3::*IFA38*::*PHS1*::*TSC13*::*ACB1* XI-5::*cas9*-*URA*	–	This study
LW21	*MAT*a *MAL2*-*8c SUC2 ura3*-*52 elo3*Δ X-2::p*MPC3*::ACC1** X-3::*IFA38*::*PHS1*::*TSC13*::*ACB1* XI-5::*cas9*-*URA*	X-4::*FAA1*::*ELO1*	This study
LW22	*MAT*a *MAL2*-*8c SUC2 ura3*-*52 elo3*Δ X-2::p*MPC3*::ACC1** X-3::*IFA38*::*PHS1*::*TSC13*::*ACB1* XI-5::*cas9*-*URA*	X-4::*FAA1*::*ELO1* XII-5::*CaKCS*::*OLE1*	This study
LW23	*MAT*a *MAL2*-*8c SUC2 ura3*-*52 elo3*Δ X-2::p*MPC3*::ACC1** X-3::*IFA38*::*PHS1*::*TSC13*::*ACB1* XI-5::*cas9*	X-4::*FAA1*::*ELO1* pYX212::*MaFAldhR*::*SciWS*::*ELO2*	This study
LW24	*MAT*a *MAL2*-*8c SUC2 ura3*-*52 elo3*Δ X-2::p*MPC3*::ACC1** X-3::*IFA38*::*PHS1*::*TSC13*::*ACB1* XI-5::*cas9*	X-4::*FAA1*::*ELO1* XII-5::*CaKCS*::*OLE1* pYX212::*MaFAldhR*::*SciWS*::*ELO2*	This study

### Cultivation of strains

*Saccharomyces cerevisiae* CEN.PK 113-5D derived strains were grown in yeast extract-peptone-dextrose (YPD) medium containing 10 g/L yeast extract (Merck Millipore), 20 g/L peptone (Difco) and 20 g/L glucose (Merck Millipore) at 30 °C and 200 RPM. Yeast strains carrying a plasmid- or integration-based *URA3* marker were selected on synthetic dextrose medium lacking uracil (SD-URA) plates, containing 6.9 g/L yeast nitrogen base (YNB) without amino acids (Formedium), 0.77 g/L complete supplement mixture (CSM) without uracil (Formedium), 20 g/L glucose and 20 g/L agar (Merck Millipore) with the pH adjusted to 5.5–6.0. To counter-select for the *URA3* marker, strains were cultivated on plates with 5-fluoroorotic acid (5-FOA), containing 6.9 g/L YNB without amino acids, 0.77 g/L CSM without uracil, 0.05 g/L uracil, 20 g/L glucose, 20 g/L agar and 1.0 g/L 5-FOA (Sigma). Strains containing the *kanMX* marker were selected on YPD plates containing 200 mg/L G418 disulfate salt (Sigma Aldrich). To select for the *natMX* marker, yeast strains were grown on YPD plates containing 100 mg/L nourseothricin (Nordic Biosite).

To monitor FA, FOH and WE synthesis, growth experiments with *S. cerevisiae* strains were performed in modified minimal medium [33], containing 7.5 g/L (NH_4_)_2_SO_4_, 14.4 g/L KH_2_PO_4_, 20 g/L glucose and with the pH adjusted to 6.5 with 5 M NaOH. In case of strains not carrying a *URA3* marker, 60 mg/L uracil (Alfa Aesar) were added to the medium. Single colonies were used to inoculate 5 mL of precultures, which were incubated for 2 days at 30 °C and 200 RPM before they were used to inoculate main cultures of 20–100 mL minimal medium in 100–500 mL shake flasks with an initial OD_600_ of 0.1. The growth rate of the strains was monitored by measuring the optical density of the cultures at 600 nm in a GENESYS™ 20 spectrophotometer (Thermo Fisher Scientific). The cells were harvested after 48 h of incubation by centrifugation at 1000×*g* for 5 min. After washing the cells with 5 mL phosphate buffer (10 mM KH_2_PO_4_, pH 7.5), the supernatant was removed, the pellet was frozen in liquid nitrogen and freeze-dried (Christ Alpha 2–4 LSC, Martin Christ Gefriertrocknungsanlagen GmbH) for 48 h.

Cultivations of *E. coli* were performed at 37 °C and 200 RPM in lysogeny broth (LB), containing 10 g/L tryptone (Merck Millipore), 5 g/L yeast extract (Merck Millipore), 10 g/L NaCl (Merck Millipore) or on plates containing additionally 20 g/L agar. *E. coli* strains carrying an *AmpR* marker were selected on LB plates containing 100 mg/L ampicillin (AppliChem).

### Construction of plasmids and strains

In this study, we investigated genes coding for KCSs from *A. thaliana* (*At*FAE) (NCBI accession no. AT4G34520), *B. napus* (*Bn*KCS) (NCBI accession no. AF490459), *C. abyssinica* (*Ca*KCS) (NCBI accession no. AY793549), *L. annua* (*La*KCS) (NCBI accession no. EU871787), *S. cerevisiae* (*Sc*Elo2p) (NCBI accession no. YCR034 W), *S. chinensis* (*Sci*FAE) (NCBI accession no. AAC49186) and *T. majus* (*Tm*KCS) (NCBI accession no. AAC49186) as well as genes coding for FADs from *Calanus hyperboreus* (*Ch*Des9-1) (NCBI accession no. AHL21604), *S. cerevisiae* (*Sc*Ole1p) (NCBI accession no. YGL055W) and *S. chinensis* (*Sci*FAD-SP) (NCBI accession no. AAA33932). In case of *Sci*FAD, a truncated version of the protein (*Sci*FAD-SP), which did not include the 31 amino acids plastid localization signal at the N-terminus, was used. We also studied two acyl-coenzyme A (CoA) desaturase-like (ADS) proteins from *A. thaliana* (*At*ADS1.2/*At*ADS1.4) (NCBI accession no. AAC49186/AAC49186). Other genes investigated in this study include one coding for a FAR from *M. aquaeolei* VT8 (*Ma*FAldhR = Maqu_2220) (NCBI accession no. YP_959486) as well as one coding for a WS from *S. chinensis* (*Sci*WS) (NCBI accession no. AF149919) and the yeast intrinsic genes *ACB1*, *ELO1*, *FAA1*, *IFA38*, *PHS1*, *TSC13*.

The heterologous genes, which were codon optimized and synthesized by Genscript, are listed in Additional file 1: Table S1. The yeast intrinsic genes *ACB1*, *ELO1*, *ELO2*, *FAA1*, *IFA38*, *OLE1*, *PHS1* and *TSC13* (NCBI accession no. NP_011551.3/NP_012339.1/NP_009963.1/NP_014962.3/NP_009717.1/NP_011460.3/NP_012438.1/NP_010269.1) were amplified based on genomic DNA of *S. cerevisiae* CEN.PK 113-5D. Genes coding for a KCS/FAE as well as for Ole1p were integrated into the region 839226–840357 of chromosome XII (XII-5). Genes coding for Faa1p and Elo1p were integrated into the region 236336–237310 of chromosome X (X-4).

Oligonucleotides used for amplification of genes included specific overhangs to promoter and terminator sequences for fusion of promoter-gene-terminator parts, using PrimeStar DNA polymerase (TaKaRa Bio). They were custom synthesized by Eurofins and are listed in Additional file 1: Table S2. Episomal plasmids containing combinations of a KCS and a FAD were constructed via modular pathway engineering based on the background plasmid pYX212 [22] (Additional file 1: Figure S1). All episomal plasmids were first assembled in *S. cerevisiae* CEN.PK 113-5D and then extracted by using the Zymoprep Yeast Plasmid Miniprep II kit (Zymo Research Corp). The plasmids were re-transformed into *E. coli* DH5α competent cells based on the method by Inoue et al. [34]. After purification of the plasmids, they were verified by restriction analysis and sequencing. Finally, the plasmids were transformed into the desired yeast strain. Integrative plasmids were constructed based on the EasyClone(-Marker Free) vector toolkit [35–37] (Additional file 1: Figure S2). Yeast competent cells were prepared and transformed with 1 µg of plasmid DNA according to the lithium acetate/single-stranded carrier DNA/polyethylene glycol method [38]. Restriction enzymes, DNA gel purification and plasmid extraction kits were purchased from Thermo Fisher Scientific.

### Construction of the background strain *S. cerevisiae* CEN.PK 113-5D *elo3*Δ X-2::p*MPC3*::ACC1** X-3::*IFA38*::*PHS1*::*TSC13*::*ACB1* XI-5::*Cas9*

The background strain *S. cerevisiae* CEN.PK 113-5D *elo3*Δ was constructed in a previous study [21] as well as the plasmid containing the double mutated ACC1** gene under control of the *S. cerevisiae MPC3* promoter and *CYC1* terminator [39]. The plasmid contains a *kanMX* marker, flanked by loxP sites, for selection, and homologous sequences guiding the integration of the fragment into the region 194944–195980 of chromosome X (X-2) [37]. The genes *ACB1*, *IFA38*, *PHS1* and *TSC13* were integrated into the region 223616–224744 of chromosome X (X-3). The *ACB1* gene is flanked by the *PGK1* promoter and *CYC1* terminator of *S. cerevisiae*, the *IFA38* gene is flanked by the *TEF1* promoter and *FBA1* terminator of *S. cerevisiae*, the *PHS1* gene is flanked by the truncated *HXT7* promoter and *TDH2* terminator of *S. cerevisiae* and the *TSC13* gene is flanked by the *TDH3* promoter and *ADH1* terminator of *S. cerevisiae*. The plasmid contains a *Kluyveromyces lactis URA3* marker, which is flanked by direct repeats [37]. All plasmids were linearized by *Not*I and the purified linear integration fragments were transformed into *S. cerevisiae* CEN.PK 113-5D *elo3*Δ by chemical transformation. To reuse the *URA3* marker, the marker was looped out by cultivating the cells overnight in YPD medium and plating them twice on 5-FOA plates before looping out of the marker was confirmed by replica plating on YPD and SD-URA plates. The *kanMX* marker was removed by transforming the cells with a plasmid containing the cre-recombinase gene under control of the *S. cerevisiae GAL1* promoter, cultivating the cells overnight in yeast extract-peptone-galactose (YPG) medium containing 10 g/L yeast extract (Merck Millipore), 20 g/L peptone (Difco) and 20 g/L galactose (Merck Millipore) and replica plating the cells on YPD and YPD + G418.

The *Cas9* gene was integrated as linear fragment under control of the *TEF1* promoter and *CYC1* terminator of *S. cerevisiae* into the region 117779–118957 of chromosome XI (XI-5). The fragment contains a *K. lactis URA3* marker, which is flanked by direct repeats. The *URA3* marker was removed in the final strains as described above.

### TLC

For preparative TLC, 20–50 mg of freeze dried cells were weighed and mixed with 10 µg lauryl laurate as well as 10 µg heptadecanol as internal standards. The extraction of lipids was performed as described previously [40] with the exception that the sample was redissolved in 50 µL chloroform/methanol (2:1, v/v). The sample was loaded onto a TLC silica gel 60 F254 plate (Merck Millipore). The mobile phase used was composed of heptane, 2-propanol and acetic acid with a ratio of 95:5:1 (v/v/v). A standard composed of 100 µg each of cholesterol, oleic acid, triolein, methyl oleate and cholesteryl oleate as well as a separate WE standard composed of 50 µg lauryl laurate were loaded onto the plate for identification of sterols, FFAs, TAGs, fatty acid methyl esters (FAMEs), SEs and WEs. The analytical standards were purchased from Nu-Check Prep, Inc with an exact weight amount. After drying of the plate, the visualization of spots was performed by spraying the plate with 0.05% 2,7-dichlorofluorescein in ethanol and exposing it to ultraviolet radiation. The spots of the TLC plate corresponding to FOHs and WEs, respectively, were scraped off with a razor blade and extracted with a mix of 3 mL hexane, 2 mL methanol and 2 mL milliQ water. The tube was vigorously vortexed, and after centrifugation at 3000 RPM for 5 min, the upper layer was transferred to a clean tube. This solution, containing the FOHs and WEs, respectively, was dried by vacuum evaporation using a miVac concentrator (Genevac) before the samples were finally dissolved in 200 µL of hexane in case of WEs and 200 µL of ethyl acetate in case of FOHs.

### Gas chromatography-mass spectrometry (GC/MS) analysis of FAMEs and WEs/

#### GC-flame ionization detector (GC-FID) analysis of FOHs

After separation of WEs from other lipids via TLC, the WE containing fraction was isolated, purified and analyzed by GC/MS (Focus GC ISQ™ single quadrupole GC; Thermo Fisher Scientific), using a ZB-50 column (L = 30 m, ID = 0.32 mm, *df* = 0.5 μm) (Phenomenex). The inlet temperature was set to 375 °C, the helium (carrier) gas flow to 1 mL/min splitless. The initial oven temperature was set to 150 °C and held for 10 min. Then the temperature was ramped to 350 °C by 7.5 °C/min and held for 10 min. The mass transfer line temperature was set to 250 °C, the ion source temperature was set to 250 °C and a full scan for m/z of 50 to 650 was performed. WEs were identified by comparison to analytical WE standards which were purchased from Nu-Check Prep, Inc. with an exact weight amount. They were dissolved in hexane and analyzed using the same protocol and column as for the samples. WEs are designated according to the nomenclature CX:Y-WE, with X indicating the total number of carbon atoms and Y indicating the total number of double bonds in the WE, e.g. C30:1, palmityl myristoleate. FOHs which were isolated and purified from TLC were analyzed via GC-FID as described previously [17]. FOH standards were purchased from Nu-Check Prep, Inc. with an exact weight amount. They were dissolved in ethyl acetate and analyzed using the same protocol and column as for the samples. FOHs are designated according to the nomenclature CX:Y-FOH, with X indicating the number of carbons and Y indicating the number of double bonds, e.g. C16:0, palmityl alcohol (hexadecanol); C16:1, palmitoleyl alcohol (hexadecenol). FAMEs were prepared and analyzed as described previously [41]. FAME standards were purchased from Sigma Aldrich. FAs are designated according to the nomenclature CX:Y, with X indicating the number of carbons and Y indicating the number of double bonds (e.g. C16:0, palmitic acid (hexadecanoic acid); C16:1-Δ9, palmitoleic acid (hexadecenoic acid)). If the position of the double bond is indicated, it is counted from the carboxyl group (Δ) and unless otherwise noted, the Z (*cis*) species (e.g. C16:1-Δ9, cis-Δ9 hexadecenoic acid).

### Determination of molecular composition of WEs

Yeast cell lysis and lipid extraction were carried out at 4 °C as previously described [42]. In short, yeast cells (~ 10 ODu) were resuspended in 1 mL 155 mM ammonium formate and lysed with 400 μL of acid-washed glass beads using a cell disruptor.

Lipids were extracted from 0.4 ODu of yeast lysate in a total of 200 μL 155 mM ammonium formate by adding 990 μL chloroform/methanol (2:1 v/v) and mixing (1400 RPM, 2 h, 4 °C) using a ThermoMixer (Eppendorf). The lower organic phase was collected after centrifugation (1000×*g*, 2 min, 4 °C). Lipid extracts were dried and kept at − 20 °C until analyzed. Lipid extracts were analyzed by positive ion mode MS^ALL^ analysis, as previously described [43], using an Orbitrap Fusion Tribrid (Thermo Fisher Scientific) equipped with a robotic nanoflow ion source, TriVersa NanoMate (Advion Biosciences). In short, lipid extracts were dissolved in 100 μL chloroform/methanol (1:2 v/v). For MS^ALL^ analysis, 10 μL lipid extract was mixed with 12.9 μL 13.3 mM ammonium formate acetate in 2-propanol and infused using a back pressure of 1.25 psi and ionization voltage of + 0.96 kV. Survey Fourier Transform MS (FTMS) data were recorded using a target resolution setting of 500,000, a max injection time of 100 ms, automated gain control of 1e5, and three microscans. Sequential FTMS^2^ data were recorded in 1.0008 amu steps across the precursor m/z range 320.3–920.8 using a quadrupole ion isolation with a width of 1.0 amu, HCD fragmentation with collision energy at 22%, max injection time of 100 ms, automated gain control of 5e4, three microscans, and a target resolution of 30,000. All FTMS data were acquired in profile mode and using an ion transfer tube temperature of 275 °C. WE molecules detected by MS^ALL^ analysis were identified and quantified using ALEX^123^ software and SAS 9.3 [43–45]. Searches of FTMS and FTMS^2^ data were done with mass tolerances of 0.0025 and 0.0050 amu, respectively, and using an in silico-generated database of ammoniated, even-chained WE molecules having a total of 28 to 56 C-atoms and 0 to 2 double bonds in their FOH and FA chains. WE molecular species detected by FTMS and FTMS^2^ analysis in all samples were annotated by molecular composition nomenclature denoting the number of C-atoms and double bonds in both the FOH and the FA chain, following the general pattern CX_1_:Y_1_-CX_2_:Y_2_, with the first part of the abbreviation referring to the FOH moiety and the second part referring to the FA moiety of the WE (e.g. C16:1-C14:0, palmityl myristoleate). The “X” in the formula indicates the number of carbon atoms in the alcohol and acid moiety, respectively, and the “Y” indicates the number of double bonds in each moiety. Data are presented as mean ± standard deviation of three biological replicates, expressed as mol% per all monitored WE molecules (calculated by normalizing the intensity of individual WE molecules to the total intensity of all identified WE molecules).

## Additional file

**Additional file 1: Table S1.** Codon optimized sequences of genes used in this study. **Table S2.** Sequences of oligonucleotides used in this study. **Table S3.** Distribution of wax ester species (mol%) in strains LW23 and LW24. The values represent the mean ± SD of three biological replicates of strains LW23 and LW24, respectively. **Figure S1.** DNA pathway assembly constructs. Genes coding for a heterologous fatty acyl reductase (FAR), a wax synthase (WS), a fatty acid elongase (FAE) or a fatty acid desaturase (FAD) were synthesized with a codon optimization for *S. cerevisiae*. The *ELO2* gene and the *OLE1* gene were amplified based on g-DNA from *S. cerevisiae* CEN.PK 113-5D. The promoter p*TPI1* and the terminator pYX212t are homologous to the respective promoter and terminator on the pYX212 plasmid. All plasmids were constructed using the modular pathway engineering strategy [22]. **Figure S2.** Integration constructs. The gene coding for *Ca*KCS was synthesized with a codon optimization for *S. cerevisiae*. The *IFA38, PHS1, TSC13*, *ACB1, FAA1, ELO1* and *OLE1* genes were amplified based on g-DNA from *S. cerevisiae* CEN.PK 113-5D. The ACC1** linear fragment, carrying a *kanMX* marker under the *Ashbya gossypii TEF*1 promoter/terminator and flanked by loxP sites, was integrated at position X-2. The *TSC13/PHS1/IFA38/ACB1* linear fragment, carrying a *Kluyveromyces lactis URA3* marker flanked by direct repeats, was integrated at position X-3. The *FAA1/ELO1* linear fragment was integrated at position X-4. The *Cas9* linear fragment was integrated at position XI-5 and the *Ca*KCS*/OLE1* linear fragment at position XII-5 in the genome. Integrative plasmids were constructed based on the EasyClone(-Marker Free) vector toolkit [35–37]. **Figure S3.** Growth behavior of strains LW21, LW22, LW23 and LW24 in minimal medium containing 20 g/L glucose. **Figure S4.** Thin layer chromatography. 1, TLC standard = 100 µg cholesterol, 100 µg oleic acid, 100 µg triolein, 100 µg methyl oleate, 100 µg cholesteryl oleate; 2, wax ester standard = 50 µg lauryl laurate (C24:0); 3, strain LW21 clone1; 4, strain LW21 clone 2; 5, strain LW21 clone 3; 6, strain LW23 clone 1; 7, strain LW23 clone 2; 8, strain LW23 clone 3. The TLC was performed as described in Materials and Methods. **Figure S5.** Thin layer chromatography. 1, TLC standard = 100 µg cholesterol, 100 µg oleic acid, 100 µg triolein, 100 µg methyl oleate, 100 µg cholesteryl oleate; 2, wax ester standard = 50 µg lauryl laurate (C24:0); 3, strain LW22 clone 1; 4, strain LW22 clone 2; 5, strain LW22 clone 3; 6, strain LW24 clone 1; 7, strain LW24 clone 2; 8, strain LW24 clone 3. The TLC was performed as described in Materials and Methods. **Figure S6.** GC-FID chromatograms of fatty alcohols (FOHs) isolated from strains LW21 (A), LW22 (B), LW23 (C) and LW24 (D). The peak labeled with (I) corresponds to the internal standard used (C17:0-FOH). A mixture of standards (c = 200 µg/mL) containing the saturated FOHs (II), C16:0-FOH; (III), C18:0-FOH; (IV), C20:0-FOH; (V), C22:0-FOH and (VI), C24:0-FOH is shown in (E). A mixture of standards (c = 200 µg/mL) containing the monounsaturated FOHs (II), C16:1-FOH; (III), C18:1-FOH; (IV), C20:1-FOH; (V), C22:1-FOH and (VI), C24:1-FOH is shown in (F). The chromatogram in (G) shows a combination of the standards (E), (c = 50 µg/mL); (F), (c = 50 µg/mL) and C17:0-FOH (c = 250 µg/mL). The strains were grown for 48 h in minimal medium containing 20 g/L glucose. **Figure S7.** GC/MS chromatograms of wax esters (WEs) isolated from strains LW21 (A), LW22 (B), LW23 (C) and LW24 (D). The peak labeled with (I) corresponds to the internal standard used (C24:0-WE). A mixture of standards (c = 10 µg/mL) containing the saturated WEs (I), C24:0-WE; (II), C30:0-WE; (III), C32:0-WE; (IV), C34:0-WE; (V), C36:0-WE, (VI), C38:0-WE; (VII), C40:0-WE and (VIII), C42:0-WE is shown in (E). A mixture of standards (c = 10 µg/mL) containing the diunsaturated WEs (III), C32:2-WE; (IV), C34:2-WE; (V), C36:2-WE; (VI), C38:2-WE; (VII), C40:2-WE; (VIII), C42:2-WE and (IX), C44:2-WE is shown in (F). The strains were grown for 48 h in minimal medium containing 20 g/L glucose. **Figure S8.** Mass spectra of selected peaks of total ion chromatograms of lipids extracted from strain LW24. Mass spectrum of the C38:2-WE (MW = 561 g/mol), eluting after 27.95 min with the specific m/z peak of 561 (A). Mass spectrum of the C40:2-WE (MW = 589 g/mol), eluting after 29.23 min with the specific m/z peak of 589 (B). Mass spectrum of the C42:2-WE (MW = 617 g/mol), eluting after 30.73 min with the specific m/z peak of 617 (C). Mass spectrum of the C44:2-WE (MW = 645 g/mol), eluting after 32.53 min with the specific m/z peak of 645 (D).

## Abbreviations

*At*: *Arabidopsis thaliana*
*Bn*: *Brassica napus*
*Ch*: *Calanus hyperboreus*
CDW: cell dry weight
*Ca*: *Crambe abyssinica*
DUWE: diunsaturated wax ester
ER: endoplasmic reticulum
ECR: enoyl-CoA reductase
FAD: fatty acid desaturase
FAE (KCS): fatty acid elongase
FAME: fatty acid methyl ester
FAS: fatty acid synthase
FA: fatty acid
FAR/FAldhR: fatty acyl reductase
FACoA: fatty acyl-CoA
FOH: fatty alcohol
FID: flame ionization detector
FT: fourier transform
FFA: free fatty acid
GC: gas chromatography
*La*: *Lunaria annua*
*Ma*: *Marinobacter aquaeolei* VT8
MS: mass spectrometry
MUFA: monounsaturated fatty acid
MUFOH: monounsaturated fatty alcohol
ODu: optical density (units)
*Sc*: *Saccharomyces cerevisiae*
*Sci*: *Simmondsia chinensis*
SD: standard deviation
SE: steryl ester
TLC: thin layer chromatography
TFAs: total fatty acids
TAG: triacylglycerol
*Tm*: *Tropaeolum majus*
(V)LCFA: (very) long-chain fatty acid
(V)LCFOH: (very) long-chain fatty alcohol
(V)LCMUFA: (very) long-chain monounsaturated fatty acid
WE: wax ester
WS: wax synthase
HCD: β-hydroxyacyl-CoA dehydratase
KCR: β-ketoacyl-CoA reductase
KCS (FAE): β-ketoacyl-CoA synthase (fatty acid elongase)
