# Supplementary material for: Increasing jojoba-like wax ester production in Saccharomyces cerevisiae by enhancing very long-chain, monounsaturated fatty acid synthesis
Source: Microb Cell Fact. 2019 Mar 11;18:49. doi: 10.1186/s12934-019-1098-9 (PMC6410506; doi:10.1186/s12934-019-1098-9)
Supplement: Supplementary file 1 — Additional file 1: Table S1. Codon optimized sequences of genes used in this study. Table S2. Sequences of oligonucleotides used in this study. Table S3. Distribution of wax ester species (mol%) in strains LW23 and LW24. The values represent the mean ± SD of three biological replicates of strains LW23 and LW24, respectively. Figure S1. DNA pathway assembly constructs. Genes coding for a heterologous fatty acyl reductase (FAR), a wax synthase (WS), a fatty acid elongase (FAE) or a fatty acid desaturase (FAD) were synthesized with a codon optimization for S. cerevisiae. The ELO2 gene and the OLE1 gene were amplified based on g-DNA from S. cerevisiae CEN.PK 113-5D. The promoter pTPI1 and the terminator pYX212t are homologous to the respective promoter and terminator on the pYX212 plasmid. All plasmids were constructed using the modular pathway engineering strategy [22]. Figure S2. Integration constructs. The gene coding for CaKCS was synthesized with a codon optimization for S. cerevisiae. The IFA38, PHS1, TSC13, ACB1, FAA1, ELO1 and OLE1 genes were amplified based on g-DNA from S. cerevisiae CEN.PK 113-5D. The ACC1** linear fragment, carrying a kanMX marker under the Ashbya gossypii TEF1 promoter/terminator and flanked by loxP sites, was integrated at position X-2. The TSC13/PHS1/IFA38/ACB1 linear fragment, carrying a Kluyveromyces lactis URA3 marker flanked by direct repeats, was integrated at position X-3. The FAA1/ELO1 linear fragment was integrated at position X-4. The Cas9 linear fragment was integrated at position XI-5 and the CaKCS/OLE1 linear fragment at position XII-5 in the genome. Integrative plasmids were constructed based on the EasyClone(-Marker Free) vector toolkit [35–37]. Figure S3. Growth behavior of strains LW21, LW22, LW23 and LW24 in minimal medium containing 20 g/L glucose. Figure S4. Thin layer chromatography. 1, TLC standard = 100 µg cholesterol, 100 µg oleic acid, 100 µg triolein, 100 µg methyl oleate, 100 µg cholesteryl oleate; 2, wax ester s [file 12934_2019_1098_MOESM1_ESM.docx]

**Table S1.** Codon optimized sequences of genes used in this study.

| **Name** | **Codon optimized sequence (5’ 🡪 3’)** | **NCBI accession no.** |
| --- | --- | --- |
| ***AtFAE*** | ATGACTTCCGTAAACGTAAAATTATTGTATAGATATGTATTGACCAACTTCTTCAACTTGTGCTTATTCCCATTAACAGCCTTCTTGGCTGGTAAAGCTAGTAGATTGACTATCAATGATTTGCATAACTTTTTATCATATTTGCAACACAACTTAATCACCGTTACTTTGTTGTTCGCTTTCACCGTATTCGGTTTGGTTTTGTACATCGTCACTAGACCTAATCCAGTCTACTTGGTAGATTATTCTTGTTACTTACCACCTCCACATTTGAAGGTTTCCGTCAGTAAAGTTATGGATATTTTCTATCAAATCAGAAAGGCTGACACTTCTTCAAGAAATGTTGCATGCGATGACCCTTCCAGTTTAGATTTCTTGAGAAAGATCCAAGAAAGATCAGGTTTGGGTGACGAAACATACTCACCAGAAGGTTTGATCCATGTTCCTCCAAGAAAGACTTTTGCTGCATCAAGAGAAGAAACAGAAAAAGTTATTATCGGTGCATTAGAAAATTTGTTCGAAAACACAAAGGTCAATCCAAGAGAAATCGGTATTTTAGTTGTCAACTCTTCAATGTTCAATCCTACACCATCTTTGTCAGCTATGGTAGTTAACACCTTTAAGTTAAGATCCAACATCAAAAGTTTCAATTTGGGTGGTATGGGTTGTTCTGCCGGTGTAATAGCTATTGATTTGGCAAAGGACTTGTTACATGTTCACAAAAACACTTATGCATTGGTCGTATCTACTGAAAATATCACACAAGGTATCTATGCCGGTGAAAACAGATCAATGATGGTATCAAATTGCTTGTTTAGAGTTGGTGGTGCCGCTATTTTGTTATCCAATAAGAGTGGTGACAGAAGAAGATCAAAGTACAAGTTGGTTCATACAGTCAGAACACACACCGGTGCTGATGACAAATCTTTTAGATGTGTTCAACAAGAAGATGACGAATCCGGTAAAATCGGTGTTTGCTTGAGTAAGGATATCACCAACGTCGCCGGTACTACATTGACCAAGAATATCGCTACTTTGGGTCCTTTGATCTTGCCATTGTCAGAAAAGTTCTTGTTTTTCGCCACATTCGTTGCTAAAAAGTTGTTGAAGGATAAGATTAAACATTACTACGTCCCTGATTTTAAGTTAGCAGTAGACCATTTCTGTATTCACGCAGGTGGTAGAGCCGTTATAGATGAATTGGAAAAGAATTTGGGTTTGTCTCCAATAGACGTTGAAGCTAGTAGATCAACTTTACACAGATTTGGTAATACCTCCAGTTCTTCAATTTGGTATGAATTGGCTTACATAGAAGCAAAGGGTAGAATGAAAAAGGGTAACAAAGCCTGGCAAATTGCTTTAGGTTCCGGTTTCAAGTGTAATAGTGCAGTATGGGTTGCCTTGAGAAACGTTAAAGCATCTGCCAATTCACCTTGGCAACATTGCATCGATAGATACCCAGTCAAGATAGATTCCGACTTATCTAAGTCAAAGACTCACGTACAAAACGGTCGTTCTTAA | AT4G34520 |
| ***BnKCS*** | ATGACATCTATTAATGTTAAATTGTTGTACCATTACGTTATTACTAATTTGTTTAATTTGTGTTTCTTTCCATTGACAGCAATTGTTGCTGGTAAAGCATATAGATTGACTATCGATGATTTGCATCATTTGTACTACTCTTACTTACAACATAATTTGATCACTATCGCTCCATTATTCGCTTTTACTGTTTTCGGTTCAGTTTTGTACATCGCAACAAGACCAAAGCCAGTTTACTTAGTTGAATACTCTTGTTACTTGCCACCAACTCATTGTAGATCATCTATCTCAAAAGTTATGGATATTTTCTATCAAGTTAGAAAGGCTGATCCATCAAGAAATGGTACATGTAACGATTCTTCATGGTTGGATTTCTTGAGAAAGATCCAAGAAAGATCAGGTTTGGGTGACGAAACTCATGGTCCAGAAGGTTTGTTACAAGTTCCACCAAGAAAAACTTTTGCTGCTGCTAGAGAAGAAACAGAACAAGTTATTATTGGTGCATTAGAAAATTTGTTTAAAAACACAAACGTTAATCCAAAAGATATTGGTATTTTAGTTGTTAATTCTTCAATGTTCAACCCAACTCCATCTTTGTCAGCTATGGTTGTTAACACTTTTAAATTGAGATCAAACGTTAGATCCTTTAATTTGGGTGGTATGGGTTGTTCTGCTGGTGTTATTGCAATTGATTTGGCTAAAGATTTGTTGCATGTTCATAAGAACACTTATGCATTGGTTGTTTCAACTGAAAACATCACATACAACATCTATGCTGGTGACAACAGATCAATGATGGTTTCAAACTGTTTGTTTAGAGTTGGTGGTGCAGCTATCTTGTTGTCTAATAAGCCAAGAGATAGAAGAAGATCAAAGTACGAATTGGTTCATACAGTTAGAACTCATACAGGTGCTGATGATAAATCTTTTAGATGTGTTCAACAAGGTGACGATGAAAACGGTAAAACTGGTGTTTCTTTATCAAAGGATATCACTGATGTTGCAGGTAGAACAGTTAAGAAAAATATCGCTACATTGGGTCCATTGATCTTGCCATTGTCTGAAAAATTGTTGTTTTTCGTTACTTTTATGGGTAAAAAGTTGTTTAAAGATAAGATTAAACATTACTACGTTCCAGATTTCAAATTGGCTATCGATCATTTCTGTATTCATGCAGGTGGTAAAGCTGTTATTGATGTTTTGGAAAAGAATTTGGGTTTGGCACCAATTGATGTTGAAGCTAGTAGATCAACTTTGCATAGATTCGGTAACACATCTTCATCTTCAATTTGGTACGAATTGGCATACATTGAAGCTAAAGGTAGAATGAAGAAAGGTAATAAGGTTTGGCAAATCGCATTGGGTTCTGGTTTTAAATGTAATTCAGCAGTTTGGGTTGCTTTGAACAACGTTAAAGCATCTACTAATTCTCCTTGGGAACATTGTATCGAAAGATATCCAGTTAAGATCGATTCTGATTCTGGTAAATCTGAAACTAGAGCACAAAATGGTAGATCATAA | AF490459 |
| ***CaKCS*** | ATGACATCTATTAATGTTAAATTGTTGTACCATTACGTTATTACTAATTTGTTTAATTTGTGTTTCTTTCCATTGACAGCAATTGTTGCTGGTAAAGCTAGTAGATTGACTATCGATGATTTGCATCATTTGTACTACTCATATTTGCAACATAACGTTATCACTATCGCTCCATTGTTCGCTTTTACTGTTTTCGGTTCTATCTTGTACATTGTTACAAGACCAAAGCCAGTTTACTTAGTTGAATACTCTTGTTACTTGCCACCAACTCAATGTAGATCATCTATCTCAAAAGTTATGGATATTTTCTATCAAGTTAGAAAGGCTGATCCTTTTAGAAATGGTACATGTGATGATTCTTCATGGTTGGATTTCTTGAGAAAGATCCAAGAAAGATCAGGTTTGGGTGACGAAACTCATGGTCCAGAAGGTTTGTTACAAGTTCCACCAAGAAAAACTTTTGCTGCTGCTAGAGAAGAAACAGAACAAGTTATTGTTGGTGCTTTGAAAAATTTGTTCGAAAACACAAAGGTTAACCCAAAAGATATTGGTATTTTGGTTGTTAATTCTTCAATGTTCAACCCAACTCCATCTTTATCAGCTATGGTTGTTAACACTTTTAAATTGAGATCAAACGTTAGATCCTTTAATTTGGGTGGTATGGGTTGTTCTGCTGGTGTTATTGCAATTGATTTGGCTAAAGATTTGTTGCATGTTCATAAAAATACTTATGCATTAGTTGTTTCAACTGAAAACATCACATACAACATCTATGCTGGTGACAACAGATCAATGATGGTTTCAAACTGTTTGTTTAGAGTTGGTGGTGCAGCTATCTTGTTGTCTAATAAGCCAAGAGATAGAAGAAGATCAAAGTACGAATTAGTTCATACAGTTAGAACTCATACAGGTGCTGATGATAAATCTTTTAGATGTGTTCAACAAGGTGACGATGAAAATGGTAAAACTGGTGTTTCTTTGTCAAAGGATATCACTGAAGTTGCAGGTAGAACAGTTAAGAAAAATATCGCTACATTGGGTCCATTGATCTTGCCATTGTCAGAAAAATTGTTGTTTTTCGTTACTTTTATGGCTAAGAAATTGTTTAAAGATAAGGTTAAGCATTACTACGTTCCAGATTTCAAATTGGCTATCGATCATTTCTGTATTCATGCAGGTGGTAGAGCTGTTATTGATGTTTTGGAAAAGAATTTGGGTTTGGCACCAATTGATGTTGAAGCTAGTAGATCAACTTTGCATAGATTCGGTAACACATCTTCATCTTCAATTTGGTACGAATTAGCATACATCGAAGCTAAAGGTAGAATGAAGAAAGGTAATAAGGTTTGGCAAATTGCATTGGGTTCTGGTTTTAAATGTAATTCAGCAGTTTGGGTTGCTTTGTCTAACGTTAAAGCATCTACTAATTCTCCTTGGGAACATTGTATCGATAGATATCCAGTTAAGATCGATTCTGATTCAGCAAAGTCTGAAACTAGAGCACAAAATGGTAGATCATAA | AY793549 |
| ***LaKCS*** | ATGACTTCTATTAATGTTAAATTGTTGTACCATTACGTTATTACTAATTTCTTTAATTTGTGTTTCTTTCCATTGACAGCTATTTTGGCTGGTAAAGCTAGTAGATTGACTACAAACGATTTGCATCATTTCTACTCTTACTTGCAACATAATTTGATTACTTTGACATTGTTGTTCGCTTTTACTGTTTTCGGTTCAGTTTTGTACTTCGTTACAAGACCAAAACCAGTTTACTTGGTTGATTACTCTTGTTACTTACCACCACAACATTTGTCAGCTGGTATCTCTAAGACTATGGAAATTTTCTATCAAATTAGAAAGTCTGATCCATTGAGAAACGTTGCATTGGATGATTCTTCTTCTTTGGATTTCTTGAGAAAGATCCAAGAAAGATCAGGTTTGGGTGACGAAACATACGGTCCAGAAGGTTTGTTCGAAATTCCACCAAGAAAGAATTTGGCTTCTGCAAGAGAAGAAACTGAACAAGTTATTAATGGTGCTTTGAAAAATTTGTTCGAAAACACAAAGGTTAACCCAAAAGAAATTGGTATTTTAGTTGTTAATTCATCTATGTTCAACCCAACTCCATCATTGTCTGCAATGGTTGTTAACACTTTTAAATTGAGATCAAACATCAAATCTTTTAATTTGGGTGGTATGGGTTGTTCAGCAGGTGTTATTGCTATTGATTTGGCAAAAGATTTGTTGCATGTTCATAAGAACACTTATGCTTTGGTTGTTTCTACTGAAAACATCACACAAAACATCTATACAGGTGACAACAGATCAATGATGGTTTCTAACTGTTTGTTTAGAGTTGGTGGTGCTGCAATTTTGTTATCAAATAAGCCAGGTGACAGAAGAAGATCAAAATATAGATTGGCTCATACTGTTAGAACTCATACAGGTGCAGATGATAAATCATTTGGTTGTGTTAGACAAGAAGAAGATGATTCTGGTAAAACAGGTGTTTCATTATCTAAGGATATCACTGGTGTTGCTGGTATCACAGTTCAAAAGAATATCACTACATTGGGTCCATTGGTTTTGCCATTGTCAGAAAAGATTTTGTTCGTTGTTACTTTTGTTGCTAAGAAATTGTTGAAGGATAAGATTAAACATTACTACGTTCCAGATTTCAAATTGGCAGTTGATCATTTCTGTATTCATGCTGGTGGTAGAGCAGTTATTGATGTTTTGGAAAAGAATTTGGGTTTGTCTCCAATTGATGTTGAAGCTAGTAGATCAACTTTGCATAGATTCGGTAACACATCATCTTCATCTATTTGGTACGAATTGGCTTACATTGAAGCAAAAGGTAGAATGAAGAAAGGTAATAAGGCTTGGCAAATTGCAGTTGGTTCAGGTTTTAAATGTAATTCTGCTGTTTGGGTTGCATTGAGAAATGTTAAAGCATCTGCAAATTCTCCTTGGGAACATTGTATCCATAAGTACCCAGTTCAAATGTACTCAGGTTCATCTAAATCTGAAACTAGAGCACAAAATGGTAGATCATAA | EU871787 |
| ***SciFAE*** | ATGAAAGCAAAAACAATAACAAATCCTGAAATACAAGTCTCAACCACTATGACCACCACCACTACCACAGCAACCTTACCAAACTTCAAATCTTCAATTAATTTGCATCACGTCAAGTTAGGTTATCATTACTTGATATCCAACGCTTTGTTTTTAGTATTCATCCCATTGTTGGGTTTGGCAAGTGCCCATTTGTCCAGTTTTTCTGCACACGATTTGTCTTTGTTGTTCGACTTGTTGAGAAGAAATTTGTTACCTGTTGTCGTTTGTTCATTTTTGTTCGTTTTGTTGGCAACTTTGCACTTTTTAACAAGACCAAGAAACGTCTATTTGGTAGATTTCGCCTGCTACAAACCACATCCTAACTTGATTACCTCTCACGAAATGTTTATGGACAGAACTTCAAGAGCTGGTTCCTTTAGTAAGGAAAACATCGAATTTCAAAGAAAGATTTTAGAAAGAGCTGGTATGGGTAGAGAAACTTATGTTCCTAAATCCGTTACCAAGGTCCCACCTGAACCAAGTATTGCTGCTGCTAGAGCTGAAGCAGAAGAAGTTATGTACGGTGCTATAGATGAAGTTTTGGAAAAGACTGGTGTAAAACCAAAGCAAATCGGTATTTTAGTTGTCAATTGTTCCTTGTTTAACCCAACACCTAGTTTATCTTCAATGATCGTTAACCATTACAAATTGCGTGGTAACATCTTGTCATACAACTTAGGTGGTATGGGTTGCTCCGCCGGTTTAATAAGTATCGATTTGGCTAAGGACTTGTTACAAGTTTACAGAAACACTTACGTCTTGGTAGTTTCTACAGAAAACATGACCTTGAACTGGTACTGGGGTAACGATAGATCAATGTTGATCACAAACTGTTTGTTCAGAATGGGTGGTGCTGCAATAATCTTGTCTAACAGATGGAGAGATAGAAGAAGATCAAAGTACCAATTGTTGCATACCGTAAGAACTCACAAAGGTGCTGATGACAAGTCCTACAGATGCGTTTTGCAACAAGAAGATGAAAACAACAAAGTTGGTGTCGCTTTGTCAAAGGACTTAATGGCCGTTGCTGGTGAAGCATTAAAGGCCAATATTACTACATTGGGTCCATTGGTCTTGCCTATGTCAGAACAATTGTTATTTTTCGCCACATTGGTAGCTAGAAAGGTTTTTAAGATGACCAACGTTAAGCCATACATCCCTGATTTCAAATTGGCCGCTAAGCATTTCTGTATCCACGCTGGTGGTAAAGCCGTTTTGGACGAATTAGAAACCAATTTGGAATTAACTCCTTGGCATTTGGAACCATCAAGAATGACTTTGTACAGATTCGGTAACACATCCAGTTCTTCATTGTGGTATGAATTAGCATACGCCGAAGCTAAAGGTAGAATTAGAAAGGGTGACAGAACTTGGATGATAGGTTTTGGTTCTGGTTTCAAATGTAATTCAGTCGTATGGAGAGCTTTAAGATCAGTTAATCCTGCTAGAGAAAAGAATCCTTGGATGGATGAAATTGAAAACTTTCCAGTACATGTTCCTAAGATTGCTCCAATAGCATCTTAA | AAC49186 |
| ***TmKCS*** | ATGTCTGGTACTAAAGCTACATCTGTTTCAGTTCCATTGCCAGATTTCAAGCAATCTGTTAATTTGAAGTATGTTAAATTGGGTTATCATTACTCAATCACTCATGCTATGTACTTGTTTTTAACACCATTGTTGTTGATCATGTCAGCACAAATTTCTACTTTTTCAATCCAAGATTTTCATCATTTGTACAACCATTTGATCTTGCATAATTTGTCTTCTTTGATTTTATGTATTGCTTTATTGTTATTTGTTTTGACATTATACTTTTTAACTAGACCAACACCAGTTTATTTGTTGAACTTCTCTTGTTACAAGCCAGATGCAATCCATAAGTGTGATAGAAGAAGATTCATGGATACTATTCGTGGTATGGGTACTTATACAGAAGAAAACATCGAATTTCAAAGAAAGGTTTTGGAAAGATCAGGTATTGGTGAATCTTCATATTTGCCACCAACAGTTTTTAAAATTCCACCAAGAGTTTACGATGCTGAAGAAAGAGCTGAAGCAGAAATGTTGATGTTTGGTGCAGTTGATGGTTTGTTCGAAAAGATTTCTGTTAAGCCAAACCAAATCGGTGTTTTGGTTGTTAACTGTGGTTTGTTTAATCCAATCCCATCTTTGTCTTCAATGATCGTTAACAGATACAAAATGCGTGGTAATGTTTTCTCTTACAATTTGGGTGGTATGGGTTGTTCTGCTGGTGTTATTTCAATTGATTTGGCAAAAGATTTGTTACAAGTTAGACCAAATTCTTATGCTTTGGTTGTTTCTTTAGAATGTATCTCTAAAAATTTGTACTTAGGTGAACAAAGATCAATGTTGGTTTCAAACTGTTTGTTTAGAATGGGTGGTGCTGCAATCTTGTTGTCTAATAAGATGTCAGATAGATGGAGATCAAAGTACAGATTGGTTCATACTGTTAGAACACATAAGGGTACTGAAGATAACTGTTTCTCTTGTGTTACAAGAAAGGAAGATTCAGATGGTAAAATCGGTATTTCTTTGTCTAAAAATTTGATGGCTGTTGCAGGTGACGCTTTGAAGACTAACATCACTACATTGGGTCCATTGGTTTTGCCAATGTCTGAACAATTGTTGTTTTTCGCTACATTGGTTGGTAAAAAGGTTTTTAAAATGAAATTGCAACCATATATTCCAGATTTCAAATTGGCATTCGAACATTTCTGTATTCATGCTGGTGGTAGAGCAGTTTTGGATGAATTGGAAAAGAATTTGAAATTATCTTCATGGCACATGGAACCATCAAGAATGTCATTGTACAGATTTGGTAATACTTCTTCATCTTCATTGTGGTATGAATTAGCTTACTCTGAAGCAAAAGGTAGAATTAAGAAAGGTGACAGAGTTTGGCAAATCGCTTTCGGTTCTGGTTTTAAATGTAATTCAGCTGTTTGGAAAGCATTAAGAAACGTTAACCCAGCAGAAGAAAAGAATCCTTGGATGGATGAAATTCATTTGTTTCCAGTTGAAGTTCCATTAAATTAA | AAL99199 |
| ***AtADS1.2*** | ATGGGTGACACTACAAAAGATGATGGTTCTTCACAATCTAAAGCTGTTAGAGGTGAAAAGAGAGCATTTTTCTTTAGAAAGTGGACTAGAATCGATATCGCTAGAGCATCAGCTGTTGGTGCTGTTCATTTGTTGTGTTTGTTGGCACCTTTTAATTACAAGTGGGAAGCATTGAGATTCGGTGTTATTTTGGCAATCGTTACTTCTTTGTCAATTACATTTTCTTACCATAGAAATTTGACACATAAATCTTTTAAATTGCCAAAATGGTTAGAATATCCATTTGCATACTCTGCTTTGTTTGCATTACAAGGTCATCCAATTGATTGGGTTTCAACTCATAGATTCCATCATCAATTCACTGATTCTGATAGAGATCCACATTCACCAATCGAAGGTTTCTGGTTCTCTCATGTTTTCTGGATCTTCGATACTTCATACATCAGAGAAAAGTGTGGTGGTAGAGATAACGTTATGGATTTGAAGCAACAATGGTTCTACAGATTTTTGAGAAACACTATCGGTTTGCATATCTTGACTTTCTGGACATTGGTTTATTTGTGGGGTGGTTTGCCATACTTAACATGTGGTGTTGGTGTTGGTGGTACTATCGGTTACAACGGTACATGGTTGATTAATTCTGCTTGTCATATTTGGGGTTCAAGAGCATGGAACACTAAGGATACTTCAAGAAATATTTGGTGGTTAGGTCCTTTTACTATGGGTGAATCTTGGCATAATAATCATCATGCTTTTGAAGCATCAGCTAGACATGGTTTGGAATGGTATCAAGTTGATTTGACATGGTATTTGATTTGTTTCTTTCAAGCATTGGGTTTAGCAACTGATGTTAAATTGCCAACAGATGCTCAAAAGAGAAAATTGGCATTTGCTAGATAA | AT1G06090 |
| ***AtADS1.4*** | ATGGGTGACAAGAATAAGGATGATTCTTCATCTCAATCAAAGGCAGTTAGAAAGGAAAAGAGAGCATTTTTGTTTAGAAAGTGGACTAGAGTTGATGTTATGAGAGTTTCTGCTGTTGGTGCAGTTCATTTGTTGTGTTTGTTGGCACCTTTTAATTACACATGGGAAGCCTTTAGATTTGCTGCAATGGTTGGTATCTCAACTAATTTGTCTATCACATTTTCATACCATAGAAATTTGACTCATAGATCCTTTAAATTGCCAAAGTGGTTAGAATACCCATTTGCTTACTCAGCATTGTTTGCTTTACAAGGTCATCCAATTGATTGGGTTTCTACTCATAGATTCCATCATCAATTCACTGATTCTGATAGAGATCCACATTCACCAATCGAAGGTTTCTGGTTCTCTCATGTTTTCTGGATCTTCGATACATCATACATCAGAGAAAAGTGTGGTGGTAGAGATAACGTTATGGATTTGAAGCAACAATGGTTCTACAGATTTTTACAAAATACTATTGGTTTGCATATCTTGACATTCTGGATTTTGGTTTATTTGTGGGGTGGTTTGCCATACTTAACTTGGTCTGTTGGTGTTGGTGGTGCAATTGGTTATCATGCTACATGGTTGATTAATTCTGCATGTCATATTTGGGGTTCAAGAGCTTGGAACACTAAGGATACTTCAAGAAATATTTGGTGGTTGGGTCCTTTTACTATGGGTGAATCTTGGCATAATAATCATCATGCATTTGAAGCATCTGCAAGACATGGTTTAGAATGGTATCAAGTTGATTTGACATGGTACTTAATTTGGTTTTTCCAAGTTTTGGGTTTAGCTACTGATGTTAAATTGCCAACAGATGCACAAAAGAGAAAAATGTCTTTAGCTAGATAA | AT1G06120 |
| ***ChDes9-1*** | ATGGAAGAAATCAACATCAAGCCTCAAGACGGTTTAAAAGAAATGGAAACAGAAGGTGACAGTGGTCATGCCTCAGACGCCTCCGACGAAGTTTTGGAATACGCTAAAAATGTTGAATCAGTCGAATTAGAACCATACGAAACTGATATAGTATGGCAAAACGTTGCCAAGTTCGTCATAATCCATGCTTTGTTTTTCTATGGTGCAACATACTTGCCTTCTATGTCATTGAACATGTGGATTTTTATGTTGATATCTACTCAAATCTCAGGTTTAGGTATTACAATGGGTGCACATAGATTATGGGCCCACAAGACATACAAGGCTAAGTTGCCATTGAGAATATTCTTGACCTTCGCTAATTCCTTAGCAGGTCAAAACTCCATATACATCTGGAGTAGAGATCATAGAACCCATCACAAATGTAGTGAAAAGATGGGTGACCCACACAATGCCAAGAGAGGTTTCTTTTTCGCTCACATGGGTTGGTTGATGGTTAGAAAACACCCTGAAGTCACAAGAGCAGGTAAAACCGTAAACATGACTGATTTGGAAAACGACAAATTGGTTATGTTACAACATAAGTACTACATCACCTCCTTCTTGTTATGTGGTTTCGTAATCCCAACTGTTTTGCCTTATTTGTTGTGGGGTGAATGCTTATATACTGCCTACTTCATGGCTATTTTTAGATACGTCATCACATTGCATGTCACCTGGTTGGTAAATTCTGCTGCACACTTCTTTGGTTACAAGCCATACGATAAGACTATAGGTCCTACAGAAAACATGTTGGTTTCATTGTTGGCTATGGGTGAAGGTTTCCATAACTATCATCACACATTTCCTTATGACTACTCCACAAGTGAATGGGGTTACACCTTTAACACTACTTCAAGAATCATTGATGCTATGGCTTCAATAGGTCAAGCCTATGATTTGAGAACCGCATCTAAAGCCACTATCGAAGCCAGAAGTGTCAGAACAGGTTTACCAGAATTAACAGCAATCTATCAAAAGAAAGCATTATAA | AHL21604 |
| ***SciFAD-SP*** | ATGGTATTCATGGCCTCATCAACTATCGGTATTACATCAAAAGAAATCCCTAACGCAAAGAAACCTCACATGCCTCCAAGAGAAGCCCATGTCCAAAAGACTCACTCCATGCCACCACAAAAGATTGAAATTTTTAAGAGTTTGGAAGGTTGGGCCGAAGAAAATGTATTGGTTCATTTGAAACCAGTTGAAAAGTGTTGGCAACCTCAAGATTTCTTGCCAGACCCTGCATCCGAAGGTTTCATGGATCAAGTTAAGGAATTAAGAGAAAGAACTAAGGAAATCCCAGATGAATATTTGGTTGTCTTAGTCGGTGACATGATTACAGAAGAAGCATTGCCTACTTACCAAACAATGTTGAACACCTTAGATGGTGTTAGAGACGAAACTGGTGCTTCCTTAACAAGTTGGGCAATTTGGACAAGAGCTTGGACCGCAGAAGAAAACAGACATGGTGACTTGTTGAACAAATATTTGTACTTAACCGGTAGAGTTGACATGAAGCAAATCGAAAAGACTATCCAATATTTGATTGGTTCCGGTATGGACCCAAGAAGTGAAAACAACCCTTATTTGGGTTTTATATATACCTCTTTCCAAGAAAGAGCAACTTTTATATCACATGGTAACACAGCCAGATTGGCTAAAGATCACGGTGACTTCCAATTAGCACAAGTATGTGGTATTATAGCTGCTGATGAAAAGAGACATGAAACCGCCTACACTAAGATCGTTGAAAAGTTGTTCGAAATCGATCCAGACGGTGCTGTCTTGGCCTTAGCTGATATGATGAGAAAGAAAGTTTCTATGCCTGCACACTTGATGTATGATGGTAAAGATGACAATTTGTTCGAAAACTACTCAGCAGTAGCCCAACAAATCGGTGTTTATACAGCAAAGGATTACGCCGACATTTTGGAACATTTGGTTAACAGATGGAAGGTCGAAAACTTGATGGGTTTATCTGGTGAAGGTCACAAGGCCCAAGATTTTGTTTGCGGTTTGGCTCCAAGAATTAGAAAATTGGGTGAAAGAGCACAATCTTTGTCAAAGCCAGTCTCTTTAGTACCTTTTTCATGGATTTTTAATAAGGAATTAAAGGTTTAA | AAA33932 |
| ***MaFAldhR* (*Maqu_2220*)** | ATGGCAATCCAACAAGTTCACCACGCAGACACAAGTTCATCCAAGGTATTAGGTCAATTAAGAGGTAAAAGAGTATTGATAACAGGTACTACAGGTTTCTTGGGTAAAGTTGTCTTAGAAAGATTGATCAGAGCTGTTCCAGATATCGGTGCAATATATTTGTTGATCAGAGGTAATAAGAGACATCCTGATGCTAGATCCAGATTCTTGGAAGAAATCGCAACATCTTCAGTTTTCGATAGATTGAGAGAAGCCGATAGTGAAGGTTTTGACGCTTTCTTGGAAGAAAGAATACACTGTGTTACTGGTGAAGTCACAGAAGCTGGTTTCGGTATCGGTCAAGAAGATTATAGAAAATTGGCCACTGAATTAGACGCTGTAATTAATTCCGCTGCAAGTGTTAACTTCAGAGAAGAATTGGATAAGGCATTAGCCATTAATACATTGTGTTTAAGAAACATAGCCGGTATGGTAGATTTGAATCCAAAGTTAGCAGTCTTGCAAGTATCCACATGCTACGTTAATGGTATGAACTCTGGTCAAGTAACCGAATCAGTTATTAAACCTGCCGGTGAAGCTGTTCCAAGATCTCCTGATGGTTTCTACGAAATAGAAGAATTAGTTAGATTGTTACAAGATAAGATAGAAGACGTCCAAGCTAGATACTCTGGTAAAGTTTTGGAAAGAAAGTTAGTCGATTTGGGTATTAGAGAAGCAAATAGATATGGTTGGTCAGACACATACACCTTTACTAAATGGTTGGGTGAACAATTGTTAATGAAGGCTTTAAACGGTAGAACATTGACCATCTTGAGACCATCTATCATCGAATCAGCATTGGAAGAACCAGCCCCTGGTTGGATAGAAGGTGTAAAAGTTGCTGATGCAATCATTTTGGCCTACGCTAGAGAAAAAGTTACCTTATTCCCAGGTAAAAGATCTGGTATAATCGATGTTATCCCTGTCGACTTAGTAGCTAACTCCATAATCTTGAGTTTAGCAGAAGCCTTGGGTGAACCAGGTAGAAGAAGAATCTATCAATGTTGCTCTGGTGGTGGTAATCCTATCAGTTTGGGTGAATTCATTGATCATTTGATGGCAGAATCTAAAGCCAACTATGCCGCTTACGACCACTTGTTTTACAGACAACCATCAAAGCCTTTCTTAGCTGTCAACAGAGCATTATTTGATTTGGTCATTTCTGGTGTAAGATTGCCATTGTCATTGACTGACAGAGTTTTGAAGTTGTTGGGTAACTCCAGAGATTTGAAGATGTTGAGAAACTTAGACACCACTCAAAGTTTGGCAACCATATTTGGTTTCTATACTGCCCCAGATTACATCTTCAGAAATGACGAATTGATGGCTTTAGCAAACAGAATGGGTGAAGTCGATAAAGGTTTGTTTCCTGTAGATGCTAGATTGATCGACTGGGAATTATATTTGAGAAAGATCCATTTGGCAGGTTTGAACAGATACGCATTGAAAGAAAGAAAAGTCTACTCATTGAAAACCGCAAGACAAAGAAAGAAAGCAGCATAA | YP_959486 |
| ***SciWS*** | ATGGAAGTAGAAAAAGAATTGAAAACCTTTAGTGAAGTCTGGATAAGTGCCATTGCCGCCGCTTGCTATTGTAGATTTGTACCTGCTGTTGCTCCACATGGTGGTGCATTGAGATTATTGTTGTTGTTGCCAGTTGTCTTGTTGTTTATTTTCTTGCCTTTGAGATTGTCTTCATTCCACTTGGGTGGTCCTACTGCATTATATTTGGTTTGGTTAGCCAACTTCAAGTTGTTGTTGTTCGCTTTCCATTTGGGTCCATTATCCAACCCTTCCTTAAGTTTGTTACACTTCATCAGTACTACATTGTTGCCAATTAAGTTTAGAGATGACCCTTCTAACGATCATGAAAAGAATAAGAGAACATTGTCATTCGAATGGAGAAAAGTAGTTTTGTTTGTTGCCAAGTTAGTCTTTTTCGCTGGTATTTTAAAGATATACGAATTCAGAAAGGATTTGCCACATTTCGTAATCTCCGTTTTGTACTGTTTCCACTTCTACTTGGGTACAGAAATAACCTTAGCTGCATCTGCAGTTATCGCCAGAGCTACTTTAGGTTTGGACTTATATCCACAATTCAATGAACCTTACTTGGCCACATCCTTACAAGATTTTTGGGGTAGAAGATGGAACTTGATGGTTAGTGACATATTGGGTTTAACCACTTATCAACCAGTCAGAAGAGTATTGTCAAGATGGGTTAGATTAAGATGGGAAGTTGCAGGTGCCATGTTGGTAGCCTTTACCGTTTCTGGTTTGATGCATGAAGTTTTCTTTTTCTATTTGACCAGAGCTAGACCTTCATGGGAAGTTACTGGTTTCTTTGTCTTACACGGTGTATGCACAGCTGTTGAAATGGTCGTTAAGAAAGCAGTCTCTGGTAAAGTAAGATTGAGAAGAGAAGTCTCAGGTGCATTAACTGTTGGTTTCGTTATGGTCACAGGTGGTTGGTTGTTTTTACCACAATTGGTTAGACATGGTGTTGATTTGAAGACAATCGACGAATACCCTGTTATGTTCAACTACACCCAAAAGAAATTGATGGGTTTGTTAGGTTGGTAA | AF149919 |

**Table S2.** Sequences of oligonucleotides used in this study.

| **Name** | **Sequence (5’ 🡪 3’) (Kozak sequence in bold; USER overhang in bold and underlined, binding part in italic; overhang in lowercase)** | **Function** |
| --- | --- | --- |
| p*TPI.AtFAE* fwd | aactacaaaaaacacatacataaact**AAAAAA***ATGACTTCCGTAAACGTAAAATTATTGTATAG* | Forward primer for amplification of *AtFAE*, containing a 5’-overhang to the *TPI* promoter in front of the Kozak sequence |
| *FBA1*t*.AtFAE* rev | aaaaaactatatcaattaatttgaattaac*TTAAGAACGACCGTTTTGTACGTGAG* | Reverse primer for amplification of *AtFAE*, containing a 5’-overhang to the *FBA1* terminator in front of the *AtFAE* stop codon |
| p*TPI.CaKCS/BnKCS* fwd | tacaaaaaacacatacataaact**AAAAAA***ATGACATCTATTAATGTTAAATTGTTGTACC* | Forward primer for amplification of *CaKCS*/*BnKCS*, containing a 5’-overhang to the *TPI* promoter in front of the Kozak sequence |
| *FBA1*t.*CaKCS*/*BnKCS*/*CgKCS*/*LaKCS* rev | aaaaaactatatcaattaatttgaa*TTAACTTATGATCTACCATTTTGTGCTCTAGTTTC* | Reverse primer for amplification of *CaKCS*/*BnKCS*/*CgKCS*/*LaKCS*, containing a 5’-overhang to the *FBA1* terminator in front of the *CaKCS*/*BnKCS*/*CgKCS*/*LaKCS* stop codon |
| p*TPI*.*CgKCS* fwd | tacaaaaaacacatacataaact**AAAAAA***ATGACTTCTATTAATGTTAAATTGTTGTACC* | Forward primer for amplification of *CgKCS*, containing a 5’-overhang to the *TPI* promoter in front of the Kozak sequence |
| p*TEF1.ELO2* fwd | tagcaatctaatctaagttttaattacaaa*ATGAATTCACTCGTTACTCAATATGCTGCTC* | Forward primer for amplification of *ELO2*, containing a 5’-overhang to the *TEF1* promoter in front of the *ELO2* start codon |
| pYX212t.*ELO2* rev | gggtcgacgcgtaagcttgtgggcccta*TTACCTTTTTCTTCTGTGTTGAGGTTTTGGTG* | Reverse primer for amplification of *ELO2*, containing a 5’-overhang to the pYX212 terminator in front of the *ELO2* stop codon |
| p*TPI.LaKCS* fwd | tacaaaaaacacatacataaact**AAAAAA***ATGACTTCTATTAATGTTAAATTGTTGTACC* | Forward primer for amplification of *LaKCS*, containing a 5’-overhang to the *TPI* promoter in front of the Kozak sequence |
| p*TPI.SciFAE* fwd | aactacaaaaaacacatacataaact**AAAAAA***ATGAAAGCAAAAACAATAACAAATCCTG* | Forward primer for amplification of *SciFAE*, containing a 5’-overhang to the *TPI* promoter in front of the Kozak sequence |
| *FBA1*t*.SciFAE* rev | aaaaaactatatcaattaatttgaattaac***TTAAGATGCTATTGGAGCAATCTTAGG*** | Reverse primer for amplification of *SciFAE*, containing a 5’-overhang to the *FBA1* terminator in front of the *SciFAE* stop codon |
| p*TPI.TmKCS* fwd | actacaaaaaacacatacataaact**AAAAAA***ATGTCTGGTACTAAAGCTACATCTG* | Forward primer for amplification of *TmKCS*, containing a 5’-overhang to the *TPI* promoter in front of the Kozak sequence |
| *FBA1*t*.TmKCS* rev | aaaaaactatatcaattaatttgaa*TTAACTTAATTTAATGGAACTTCAACTGGAAAC* | Reverse primer for amplification of *TmKCS*, containing a 5’-overhang to the *FBA1* terminator in front of the *TmKCS* stop codon |
| p*PGK1.AtADS1.2* fwd | ttatctactttttacaacaaatataac**AAAAAA***ATGGGTGACACTACAAAAGATGATG* | Forward primer for amplification of *AtADS1.2*, containing a 5’-overhang to the *PGK1* promoter in front of the Kozak sequence |
| *ADH1*t*.AtADS1.2* rev | aataaaaatcataaatcataagaaattcgc*TTATCTAGCAAATGCCAATTTTCTCTTTTGAG* | Reverse primer for amplification of *AtADS1.2*, containing a 5’-overhang to the *ADH1* terminator in front of the *AtADS1.2* stop codon |
| p*PGK1.AtADS1.4* fwd | ttatctactttttacaacaaatataac**AAAAAA***ATGGGTGACAAGAATAAGGATGATTC* | Forward primer for amplification of *AtADS1.4*, containing a 5’-overhang to the *PGK1* promoter in front of the Kozak sequence |
| *ADH1*t*.AtADS1.4* rev | aataaaaatcataaatcataagaaattcgc*TTATCTAGCTAAAGACATTTTTCTCTTTTGTGC* | Reverse primer for amplification of *AtADS1.4*, containing a 5’-overhang to the *ADH1* terminator in front of the *AtADS1.2* stop codon |
| p*PGK.ChDes9-1* fwd | ttatctactttttacaacaaatataac**AAAACA***ATGGAAGAAATCAACATCAAGC* | Forward primer for amplification of *ChDes9-1*, containing a 5’-overhang to the *PGK1* promoter in front of the Kozak sequence |
| *ADH1*t*.ChDes9-1* rev | aataaaaatcataaatcataagaaattcgc*TTATAATGCTTTCTTTTGATAGATTGCTG* | Reverse primer for amplification of *ChDes9-1*, containing a 5’-overhang to the *ADH1* terminator in front of the *ChDes9-1* stop codon |
| p*PGK.OLE1* fwd | ttatctactttttacaacaaatataac**AAAAAA***ATGCCAACTTCTGGAACTACTATTGAATTG* | Forward primer for amplification of *OLE1*, containing a 5’-overhang to the *PGK1* promoter in front of the Kozak sequence |
| *ADH1*t*.OLE1* rev | aataaaaatcataaatcataagaaattcgc*TTAAAAGAACTTACCAGTTTCGTAGATTTCAC* | Reverse primer for amplification of *OLE1*, containing a 5’-overhang to the *ADH1* terminator in front of the *OLE1* stop codon |
| p*PGK.SciFAD-SP* fwd | ttatctactttttacaacaaatataac**AAAAAA***ATGGTATTCATGGCCTCATCAAC* | Forward primer for amplification of *SciFAD-SP*, containing a 5’-overhang to the *PGK1* promoter in front of the Kozak sequence |
| *ADH1*t*.SciFAD-SP* rev | aataaaaatcataaatcataagaaattcgc*TTAAACCTTTAATTCCTTATTAAAAATCCATG* | Reverse primer for amplification of *SciFAD-SP*, containing a 5’-overhang to the *ADH1* terminator in front of the *SciFAD-SP* stop codon |
| p*TPI* fwd | *GTTTAAAGATTACGGATATTTAACTTACTTAGAATAATG* | Forward primer for amplification of the *TPI* promoter |
| p*TPI* rev | ca*TTTTAGTTTATGTATGTGTTTTTTGTAG* | Reverse primer for amplification of the *TPI* promoter |
| *FBA1*t fwd | *GTTAATTCAAATTAATTGATATAGTTTTTTAATGAG* | Forward primer for amplification of the *FBA1* terminator |
| *FBA1*t rev | *AGTAAGCTACTATGAAAGACTTTACAAAGAAC* | Reverse primer for amplification of the *FBA1* terminator |
| *CYC1*t fwd | *GATACCGTCGACCTCGAGTCATGTAATTAGTTATGTC* | Forward primer for amplification of the *CYC1* terminator |
| *CYC1*t rev | *GGGTACCGGCCGCAAATTAAAGCCTTCGAGCGTCC* | Reverse primer for amplification of the *CYC1* terminator |
| p*TDH3* fwd | c*TCGAGTTTATCATTATCAATACTGCCATTTC* | Forward primer for amplification of the *TDH3* promoter |
| p*TDH3* rev | *GTTTGTTTATGTGTGTTTATTCGAAACTAAGTTCTTGGTG* | Reverse primer for amplification of the TDH3 promoter |
| p*HXT7* fwd | gtattctttgaaatggcagtattgataatgataaactcgag*CTCGTAGGAACAATTTCG* | Forward primer for amplification of the *HXT7* promoter |
| p*HXT7* rev | cat*TTTTTGATTAAAATTAAAAAAACTTTTTGTTTTTGTG* | Reverse primer for amplification of the *HXT7* promoter |
| *TDH2*t fwd | *ATTTAACTCCTTAAGTTACTTTAATGATTTAGTTTTTA* | Forward primer for amplification of the *TDH2* terminator |
| *TDH2*t rev | *GCGAAAAGCCAATTAGTGTGATAC* | Reverse primer for amplification of the *TDH2* terminator |
| *ADH1*t fwd | *GCGAATTTCTTATGATTTATGATTTTTATTATTAAATAAG* | Forward primer for amplification of the *ADH1* terminator |
| *ADH1*t rev | *GCATATCTACAATTGGGTGAAATGGGGAGCGATTTG* | Reverse primer for amplification of the *ADH1* terminator |
| p*PGK1* fwd | *CGCACAGATATTATAACATCTGCACAATAGG* | Forward primer for amplification of the *PGK1* promoter |
| p*PGK1* rev | cat*TTTGTTATATTTGTTGTAAAAAGTAGATAATTAC* | Reverse primer for amplification of the *PGK1* promoter |
| p*TEF1* fwd | *ATAGCTTCAAAATGTTTCTACTCCTTTTTTACTC* | Forward primer for amplification of the *TEF1* promoter |
| p*TEF1* rev | *TTTGTAATTAAAACTTAGATTAGATTGCTATGC* | Reverse primer for amplification of the *TEF1* promoter |
| pYX212t fwd | *TAGGGCCCACAAGCTTACGCGTCGACCCGGGTATCC* | Forward primer for amplification of the pYX212 terminator |
| pYX212t rev | *GCCGTAAACCACTAAATCGGAACCCTAAAGG* | Reverse primer for amplification of the pYX212 terminator |
| pYX212t seq | *TAGTCAGGCACATCATACGG* | Sequencing primer |
| p*TDH3* seq | *ACGGTAGGTATTGATTGTAATTC* | Sequencing primer |
| p*TEF1* seq | *TGACCTCCCATTGATATTTAAG* | Sequencing primer |
| p*TPI* seq | *TGCAACATTTACTATTTTCCCTTCTTACG* | Sequencing primer |
| *FBA1*t seq | *TTCAGAAGAAAAGAGCCGACC* | Sequencing primer |
| *ADH1*t seq | *TCAATAAGAGCGACCTCATGCTATAC* | Sequencing primer |
| p*PGK1* seq | *TTTCAAGTTCTTAGATGCTTTCTTTTTCTC* | Sequencing primer |
| *CYC1*t fwd seq | *AATAGGGACCTAGACTTCAG* | Sequencing primer |
| p*HXT7* fwd seq | *TTCTTCATTTGCAGCTATTG* | Sequencing primer |
| *TDH2*t rev seq | *TCCCTGAGGAATCTTTAATAC* | Sequencing primer |
| p*TDH3* rev seq | *TGATTGTAATTCTGTAAATC* | Sequencing primer |
| *ELO1* GP2F (USER) (p*TEF1*) | **ATCTGTCAUAAAACA***ATGGTAAGTGATTGGAAAAATTTTTGCCTC* | Forward primer for amplification of *ELO1*, containing a 5’- USER overhang in front of the Kozak sequence |
| *ELO1* GV2R (USER) (p*TEF1*) | **CACGCGAU***TTAATTGTTTTTGTTGATCTTCTTCTTACCAC* | Reverse primer for amplification of *ELO1*, containing a 5’- USER overhang in front of the *ELO1* stop codon |
| *FAA*1 GP1F (USER) (p*HXT1*) | **AGTGCAGGUAAAACA***ATGGTTGCTCAATATACCGTTCCAG* | Forward primer for amplification of *FAA1*, containing a 5’- USER overhang in front of the Kozak sequence |
| *FAA1* GV1R (USER) (p*HXT1*) | **CGTGCGAU***TTAAGACGAACTATAAACGGCGTCAAC* | Reverse primer for amplification of *FAA1*, containing a 5’- USER overhang in front of the *FAA1* stop codon |
| *CaKCS* GP1F (USER) (p*TPI*) | **AGTGCAGGUAAAACA***ATGACATCTATTAATGTTAAATTGTTGTAC* | Forward primer for amplification of *CaKCS*, containing a 5’- USER overhang in front of the Kozak sequence |
| *CaKCS* GP2F (USER) (p*PGK1*) | **ATCTGTCAUAAAACA***ATGACATCTATTAATGTTAAATTGTTGTAC* | Forward primer for amplification of *CaKCS*, containing a 5’- USER overhang in front of the Kozak sequence |
| *CaKCS*/*LaKCS* GV1R (USER) (p*TPI*) | **CGTGCGAU***TTATGATCTACCATTTTGTGCTCTAGTTTC* | Reverse primer for amplification of *CaKCS*/*LaKCS*, containing a 5’- USER overhang in front of the *CaKCS*/*LaKCS* stop codon |
| *CaKCS*/*LaKCS* GV2R (USER) (p*PGK1*) | **CACGCGAU***TTATGATCTACCATTTTGTGCTCTAGTTTC* | Reverse primer for amplification of *CaKCS*/*LaKCS*, containing a 5’- USER overhang in front of the the *CaKCS*/*LaKCS* stop codon |
| *ELO2* GP1F (USER) (p*TPI*) | **AGTGCAGGUAAAACA***ATGAATTCACTCGTTACTCAATATGC* | Forward primer for amplification of *ELO2*, containing a 5’- USER overhang in front of the Kozak sequence |
| *ELO2* GV1R (USER) (p*TPI*) | **CGTGCGAU***TTACCTTTTTCTTCTGTGTTGAGGTTTTG* | Reverse primer for amplification of *ELO2*, containing a 5’- USER overhang in front of the *ELO2* stop codon |
| *LaKCS* GP1F (USER) (p*TPI*) | **AGTGCAGGUAAAACA***ATGACTTCTATTAATGTTAAATTGTTGTAC* | Forward primer for amplification of *LaKCS*, containing a 5’- USER overhang in front of the Kozak sequence |
| *LaKCS* GP2F (USER) (p*PGK1*) | **ATCTGTCAUAAAACA***ATGACTTCTATTAATGTTAAATTGTTGTAC* | Forward primer for amplification of *LaKCS*, containing a 5’- USER overhang in front of the Kozak sequence |
| *AtADS1.2* GP2F (USER) (p*PGK1*) | **ATCTGTCAUAAAACA***ATGGGTGACACTACAAAAGATG* | Forward primer for amplification of *AtADS1.2*, containing a 5’- USER overhang in front of the Kozak sequence |
| *AtADS1.2* GV2R (USER) (p*PGK1*) | **CACGCGAU***TTATCTAGCAAATGCCAATTTTCTCTTTTG* | Reverse primer for amplification of *AtADS1.2*, containing a 5’- USER overhang in front of the *AtADS1.2* stop codon |
| *ChDes9-1* GP2F (USER) (p*PGK1*) | **ATCTGTCAUAAAACA***ATGGAAGAAATCAACATCAAGC* | Forward primer for amplification of *ChDes9-1*, containing a 5’- USER overhang in front of the Kozak sequence |
| *ChDes9-1* GV2R (USER) (p*PGK1*) | **CACGCGAU***TTATAATGCTTTCTTTTGATAGATTGCTG* | Reverse primer for amplification of *ChDes9-1*, containing a 5’- USER overhang in front of the *ChDes9-1* stop codon |
| *OLE1* GP2F (USER) (p*PGK1*) | **ATCTGTCAUAAAACA***ATGCCAACTTCTGGAACTACTATTG* | Forward primer for amplification of *OLE1*, containing a 5’- USER overhang in front of the Kozak sequence |
| *OLE1* GV2R (USER) (p*PGK1*) | **CACGCGAU***TTAAAAGAACTTACCAGTTTCGTAGATTTC* | Reverse primer for amplification of *OLE1*, containing a 5’- USER overhang in front of the *OLE1* stop codon |
| *SciFAD-SP* GP2F (USER) (p*PGK1*) | **ATCTGTCAUAAAACA***ATGGTATTCATGGCCTCATCAAC* | Forward primer for amplification of *SciFAD-SP*, containing a 5’- USER overhang in front of the Kozak sequence |
| *SciFAD-SP* GV2R (USER) (p*PGK1*) | **CACGCGAU***TTAAACCTTTAATTCCTTATTAAAAATCCATGAAAAAG* | Reverse primer for amplification of *SciFAD-SP*, containing a 5’- USER overhang in front of the *SciFAD-SP* stop codon |
| p*TDH3*/p*HXT7* PG1R (USER) | **ACCTGCACU***TTTGTTTGTTTATGTGTGTTTATTCGAAAC* | Primer for amplification of the *TDH3*/*HXT7* double promoter, containing a 5’- USER overhang |
| p*TDH3*/p*HXT7* PG2R (USER) | **ATGACAGAU***TTTTTGATTAAAATTAAAAAAACTTTTTGTTTTTGTG* | Primer for amplification of the *TDH3*/*HXT7* double promoter, containing a 5’- USER overhang |
| p*TPI*/p*PGK1* PG1R (USER) | **ACCTGCACU***TTTTAGTTTATGTATGTGTTTTTTGTAGTTATAGATTTAAG* | Primer for amplification of the *TPI*/*PGK1* double promoter, containing a 5’- USER overhang |
| p*TPI*/p*PGK1* PG2R (USER) | **ATGACAGAU***TTTGTTATATTTGTTGTAAAAAGTAGATAATTACTTCC* | Primer for amplification of the *TPI*/*PGK1* double promoter, containing a 5’- USER overhang |
| p*TPI* fwd (overhang p*PGK1*) | tgtgcagatgttataatatctgtgcgt*GTTTAAAGATTACGGATATTTAACTTAC* | Primer for amplification of the *TPI* promoter, containing a 5’-overhang to the *PGK1* promoter |
| p*PGK1* fwd (overhang p*TPI*) | taagttaaatatccgtaatctttaaac*ACGCACAGATATTATAACATCTGCA* | Primer for amplification of the *PGK1* promoter, containing a 5’-overhang to the *TPI* promoter |
| p*HXT1*/p*TEF1* PG1R (USER) | **ACCTGCACU***GATTTTACGTATATCAACTAGTTGACGATTATG* | Primer for amplification of the *HXT1*/*TEF1* double promoter, containing a 5’- USER overhang |
| p*HXT1*/p*TEF1* PG2R (USER) | **ATGACAGAU***TTTGTAATTAAAACTTAGATTAGATTGCTATGC* | Primer for amplification of the *HXT1*/*TEF1* double promoter, containing a 5’- USER overhang |
| p*HXT1* fwd (overhang p*TEF1*) | aggagtagaaacattttgaagctat*GGCCACAATGAAACTTCAATTCATATCG* | Primer for amplification of the *HXT11* promoter, containing a 5’-overhang to the *TEF1* promoter |
| p*TEF1* fwd (overhang p*HXT1*) | tcgatatgaattgaagtttcattgtggcc*ATAGCTTCAAAATGTTTCTACTCCTTTTTTAC* | Primer for amplification of the *TEF1* promoter, containing a 5’-overhang to the *HXT1* promoter |
| *ECR* fwd (p*TDH3*) | aataaacacacataaacaaa**CAAA***ATGCCTATCACCATAAAAAGCCGC* | Forward primer for amplification of the *ECR*, containing a 5’-overhang to the *TDH3* promoter in front of the Kozak sequence |
| *ECR* rev (USER) | **CGTGCGAU***TCAAAATACAAATGGAATCAAGAATGCTC* | Reverse primer for amplification of *ECR*, containing a 5’- USER overhang |
| *HCD* fwd (p*HXT7*) | ttttttaattttaat**CAAAAA***ATGTCAAAAAAACTTGCGTCACCATTGTC* | Forward primer for amplification of the *HCD*, containing a 5’-overhang to the *HXT7* promoter in front of the Kozak sequence |
| *HCD* rev (USER) | **AGTGCAGGU***TCAAATTAGTTTCTTCCCGAAAGAGG* | Reverse primer for amplification of *HCD*, containing a 5’- USER overhang |
| *ECR*/p*TDH3*/p*HXT7*/*HCD* GV1R (USER) | **CGTGCGAU***TCAAAATACAAATGGAATCAAGAATGCTC* | Primer for amplification of the *ECR*/p*TDH3*/p*HXT7*/*HCD* fragment, containing a 5’- USER overhang |
| *ECR*/p*TDH3*/p*HXT7*/*HCD* GP1F (USER) | **AGTGCAGGU***TCAAATTAGTTTCTTCCCGAAAGAGG* | Primer for amplification of the *ECR*/p*TDH3*/p*HXT7*/*HCD* fragment, containing a 5’- USER overhang |
| *TDH2*t fwd (USER) | **ACCTGCACU***ATTTAACTCCTTAAGTTACTTTAATGATTTAG* | Primer for amplification of the *TDH2*/*FBA1* double terminator, containing a 5’- USER overhang |
| *TDH2*t rev (t*FBA1*) | tgtaaagtctttcatagtagcttact*GCGAAAAGCCAATTAGTGTGATAC* | Primer for amplification of the *TDH2* terminator, containing a 5’- overhang to the *FBA1* terminator |
| *FBA1*t rev (t*TDH2*) | *tcacactaattggcttttcgcAGTAAGCTACTATGAAAGACTTTACAAAG* | Primer for amplification of the *FBA1* terminator, containing a 5’- overhang to the *FBA1* terminator |
| *FBA1*t fwd (USER) | **ATGACAGAU***GTTAATTCAAATTAATTGATATAGTTTTTTAATGAG* | Primer for amplification of the *TDH2*/*FBA1* double terminator, containing a 5’- USER overhang |
| *KCR* fwd (p*TEF1*) | atctaatctaagttttaatta**CAAA***ATGACTTTTATGCAACAGCTTCAAG* | Forward primer for amplification of *KCR*, containing a 5’- overhang to the *TEF1* promoter |
| *KCR* rev (USER) | **ATCTGTCAU***CTATTCCTTTTTAACCTGTCTTGCG* | Reverse primer for amplification of *KCR*, containing a 5’- USER overhang |
| *ACB1* fwd (p*PGK1*) | tttttacaacaaatataa**CAAA***ATGGTTTCCCAATTATTCGAAGAAAAAG* | Forward primer for amplification of *ACB1*, containing a 5‘ overhang to the *PGK1* promoter |
| *ACB1* rev (USER) | **CACGCGAU***CTAAGAGGAGTACTTGGCAATCAG* | Reverse primer for amplification of *ACB1*, containing a 5‘ – USER overhang |
| *KCR*/p*TEF1*/p*PGK1*/*ACB1* GP2F (USER) | **ATCTGTCAU***CTATTCCTTTTTAACCTGTCTTGCG* | Primer for amplification of the *KCR*/p*TEF1*/p*PGK1*/*ACB1* fragment, containing a 5’- USER overhang |
| *KCR*/p*TEF1*/p*PGK1*/*ACB1* GV2R (USER) | **CACGCGAU***CTAAGAGGAGTACTTGGCAATCAG* | Primer for amplification of the *KCR*/p*TEF1*/p*PGK1*/*ACB1* fragment, containing a 5’- USER overhang |
| *Cas9* cassette 1 fwd (XI-5) | *GCGGAGAAGTCGTTGATAGC* | Forward primer for amplification of part 1 of the *Cas9* cassette |
| *Cas9* cassette 1 rev | *AGTGATGGTCTCTTCTGATTTGCG* | Reverse primer for amplification of part 1 of the *Cas9* cassette |
| *Cas9* cassette 2 fwd | *TTCTGCGAGTGAACACGGAG* | Forward primer for amplification of part 2 of the *Cas9* cassette |
| *Cas9* cassette 2 rev (XI-5) | *GATCATAGATCCGGCACTTAGAG* | Reverse primer for amplification of part 2 of the *Cas9* cassette |
| *ADH1*t_easyclone | GAAATTCGCTTATTTAGAAGTGTC | Sequencing primer |
| *CYC1*t_easyclone | CTCCTTCCTTTTCGGTTAGAG | Sequencing primer |
| X-2 up fwd | *GAAATTTTCATTATGTGTTAGAGAGAGACG* | Forward primer binding in chromosome X in front of the insertion region of the X-2 insertion fragment to verify the integration of *ACC1*** at the right site in the genome |
| *ADH1*t-p*MPC3* rev | *ACTATTCCCATCGTTGGTAGATACGTTGTTGAC* | Reverse primer binding in the *ADH1* terminator/*MPC3* promoter region in the X-2 insertion fragment to verify the integration of *ACC1*** at the right site in the genome |
| *ADH1*t-p*MPC3* fwd | *GTATCTACCAACGATGGGAATAGTGCCTTAGC* | Forward primer binding in the *ADH1* terminator/*MPC3* promoter region in front of *ACC1*** in the X-2 insertion fragment to verify the integration of the complete *ACC1*** gene |
| *ACC1***-*CYC1*t rev | *GATAAAGAAAAATTGTTGAAGACTTTGAAATAAATCCGCTCTAACCGA* | Reverse primer binding in the *CYC1* terminator/end of *ACC1*** in the X-2 insertion fragment to verify the integration of the complete *ACC1*** gene |
| *ACC1***-*CYC1*t fwd | *GACTTTGAAATAAATCCGCTCTAACCGAAAAGG* | Forward primer binding in the *CYC1* terminator/end of *ACC1*** in the X-2 insertion fragment to verify the integration of *ACC1*** at the right site in the genome |
| X-2 down rev | *CTTTGTAGAACAGCTCTCTCTTAAACAC* | Reverse primer binding in chromosome X behind of the insertion region of the X-2 insertion fragment to verify the integration of *ACC1*** at the right site in the genome |
| X-3 up fwd | *TGACGAATCGTTAGGCACAG* | Forward primer binding in chromosome X in front of the insertion region of the X-3 insertion fragment to verify the integration of the  *ECR*/*HCD*/*KCR*/*ACBP* cassette at the right site in the genome |
| X-3 up rev | *GTTGACACTTCTAAATAAGCGAATTTC* | Reverse primer binding in the *ADH1* terminator region of the X-3 insertion fragment to verify the integration of the *ECR*/*HCD*/*KCR*/*ACBP* cassette at the right site in the genome |
| X-3 down fwd | *CCTGCAGGACTAGTGCTGAG* | Forward primer binding in the X-3 insertion fragment to verify the integration of the *ECR*/*HCD*/*KCR*/*ACBP* cassette at the right site in the genome |
| X-3 down rev | *CCGTGCAATACCAAAATCG* | Reverse primer binding in chromosome X behind the insertion region of the X-3 insertion fragment to verify the integration of the *ECR*/*HCD*/*KCR*/*ACBP* cassette at the right site in the genome |
| X-4 up fwd | *CTCACAAAGGGACGAATCCT* | Forward primer binding in chromosome X in front of the insertion region of the X-4 insertion fragment to verify the integration of *FAA1* and *ELO1* at the right site in the genome |
| X-4 up rev | *GTTGACACTTCTAAATAAGCGAATTTC* | Reverse primer binding in the *ADH1* terminator region of the X-4 insertion fragment to verify the integration of *FAA1* and *ELO1* at the right site in the genome |
| X-4 down fwd | *CCTGCAGGACTAGTGCTGAG* | Forward primer binding in the X-4 insertion fragment to verify the integration of *FAA1* and *ELO1* at the right site in the genome |
| X-4 down rev | *GACGGTACGTTGACCAGAG* | Reverse primer binding in chromosome X behind the insertion region of the X-4 insertion fragment to verify the integration of *FAA1* and *ELO1* at the right site in the genome |
| XI-5 up fwd | ***CTCAATGATCAAAATCCTGAATGCA*** | Forward primer binding in chromosome XI in front of the insertion region of the XI-5 insertion fragment to verify the integration of the *Cas9* gene at the right site in the genome |
| *Cas9* integration verification primer 1 | ***TCGGCGAATTTTCTGTCCTC*** | Reverse primer binding in the *URA* marker region of the *Cas9* integration cassette to verify the integration of the *Cas9* gene at the right site in the genome |
| *Cas9* integration verification primer 2 | *ACCTCATCATTAAGCTTCCCAAGTAC* | Forward primer binding in the *Cas9* region of the *Cas9* integration cassette to verify the integration of the *Cas9* gene at the right site in the genome |
| XI-5 down rev | ***GCATGGTCACCGCTATCAGC*** | Forward primer binding in chromosome XI behind the insertion region of the XI-5 insertion fragment to verify the integration of the *Cas9* gene at the right site in the genome |
| XII-5 up fwd | ***CCACCGAAGTTGATTTGCTT*** | Forward primer binding in chromosome XII in front of the insertion region of the XII-5 insertion fragment to verify the integration of the different *KCS* and *FAD* combinations at the right site in the genome |
| XII-5 up rev | ***GTTGACACTTCTAAATAAGCGAATTTC*** | Reverse primer binding in the *ADH1* terminator region of the XII-5 insertion fragment to verify the integration of the different *KCS* and *FAD* combinations at the right site in the genome |
| XII-5 down fwd | ***CCTGCAGGACTAGTGCTGAG*** | Forward primer binding in the XII-5 insertion fragment to verify the integration of the different *KCS* and *FAD* combinations at the right site in the genome |
| XII-5 down rev | ***GTGGGAGTAAGGGATCCTGT*** | Reverse primer binding in chromosome XII behind the insertion region of the XII-5 insertion fragment to verify the integration of the different *KCS* and *FAD* combinations at the right site of the genome |

**Table S3.** Distribution of wax ester species (mol%) in strains LW23 and LW24. The values represent the mean $\pm$ SD of three biological replicates of strains LW23 and LW24, respectively.

| **Wax ester species**  **(CX:Y;**  **X, number of carbon atoms;**  **Y, number of double bonds)** | **LW23** | **LW24** |
| --- | --- | --- |
| **C30:1** | 0.00 ± 0.00 | 0.03 ± 0.04 |
| **C32:1** | 2.05 ± 0.15 | 0.48 ± 0.06 |
| **C32:2** | 0.00 ± 0.00 | 0.24 ± 0.10 |
| **C34:0** | 25.30 ± 1.07 | 0.23 ± 0.03 |
| **C34:1** | 17.24 ± 0.86 | 2.70 ± 0.64 |
| **C34:2** | 1.76 ± 0.07 | 3.79 ± 1.34 |
| **C36:1** | 14.60 ± 0.15 | 3.20 ± 0.61 |
| **C36:2** | 1.65 ± 0.21 | 7.79 ± 1.85 |
| **C38:1** | 14.31 ± 0.25 | 6.36 ± 1.15 |
| **C38:2** | 0.47 ± 0.08 | 6.31 ± 0.77 |
| **C40:1** | 21.04 ± 1.47 | 14.01 ± 1.55 |
| **C40:2** | 0.31 ± 0.02 | 10.29 ± 0.41 |
| **C42:1** | 1.20 ± 0.09 | 17.43 ± 0.63 |
| **C42:2** | 0.04 ± 0.02 | 18.26 ± 4.18 |
| **C44:1** | 0.03 ± 0.02 | 3.69 ± 0.97 |
| **C44:2** | 0.00 ± 0.00 | 4.33 ± 2.80 |
| **C46:1** | 0.00 ± 0.00 | 0.39 ± 0.08 |
| **C46:2** | 0.00 ± 0.00 | 0.46 ± 0.22 |


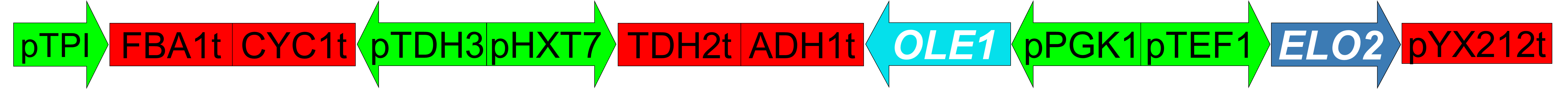

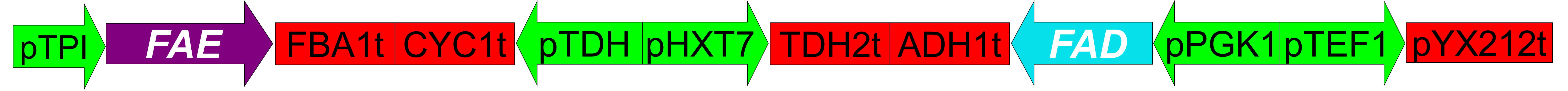

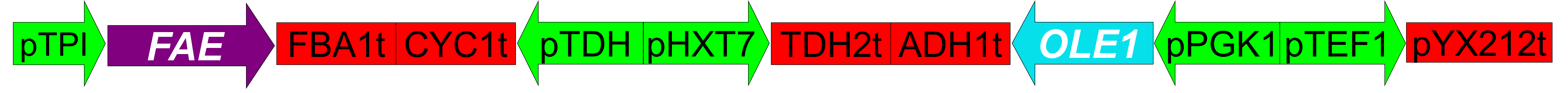

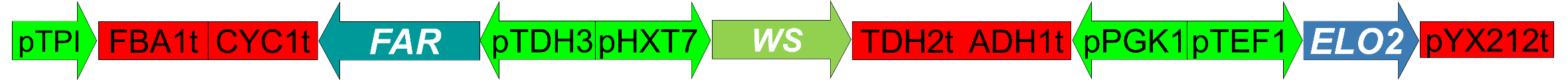


**Figure S1.** DNA pathway assembly constructs. Genes coding for a heterologous fatty acyl reductase (FAR), a wax synthase (WS), a fatty acid elongase (FAE) or a fatty acid desaturase (FAD) were synthesized with a codon optimization for *S. cerevisiae*. The *ELO2* gene and the *OLE1* gene were amplified based on g-DNA from *S. cerevisiae* CEN.PK 113-5D. The promoter pTPI and the terminator pYX212t are homologous to the respective promoter and terminator on the pYX212 plasmid. All plasmids were constructed using the modular pathway engineering strategy (Zhou et al., 2012).

**
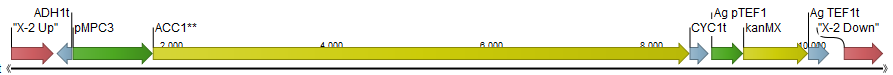
**

**
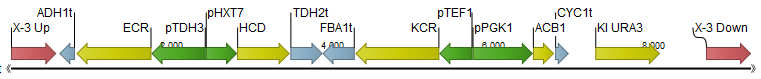
**

**
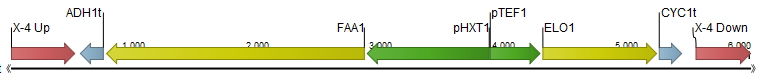
**

**
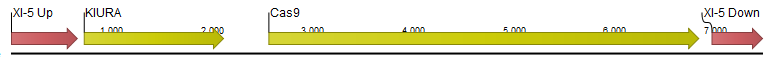
**

**
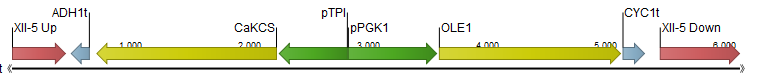
**

**Figure S2.** Integration constructs. The gene coding for *Ca*KCS was synthesized with a codon optimization for *S. cerevisiae*. The *ECR*, *HCD*, *KCR*, *ACB1*, *FAA1,* *ELO1* and *OLE1* genes were amplified based on g-DNA from *S. cerevisiae* CEN.PK 113-5D. The *ACC1*** linear fragment, carrying a *kanMX* marker under the *Ashbya gossypii TEF*1 promoter/terminator and flanked by loxP sites, was integrated at position X-2. The *ECR/HCD/KCR/ACB1* linear fragment, carrying a *Kluyveromyces lactis URA3* marker flanked by direct repeats, was integrated at position X-3. The *FAA1/ELO1* linear fragment was integrated at position X-4. The *Cas9* linear fragment was integrated at position XI-5 and the *Ca*KCS*/OLE1* linear fragment at position XII-5 in the genome. Integrative plasmids were constructed based on the EasyClone(-Marker Free) vector toolkit (Jensen et al., 2014; Jessop-Fabre et al., 2016; Mikkelsen et al., 2012).


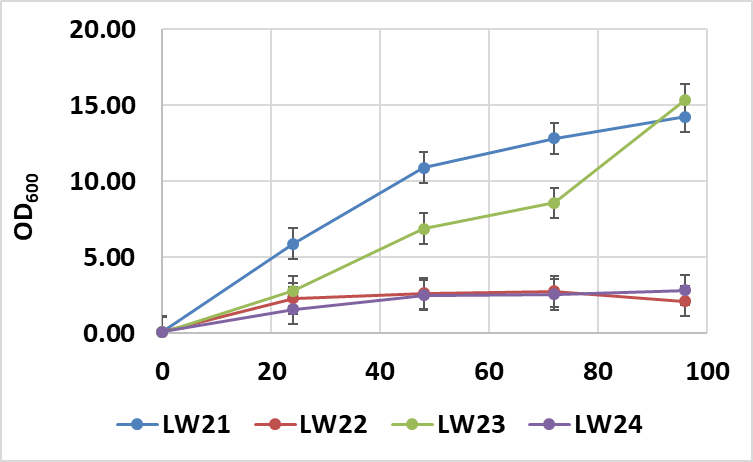


**Figure S3.** Growth behavior of strains LW21, LW22, LW23 and LW24 in minimal medium containing 20 g/L glucose.


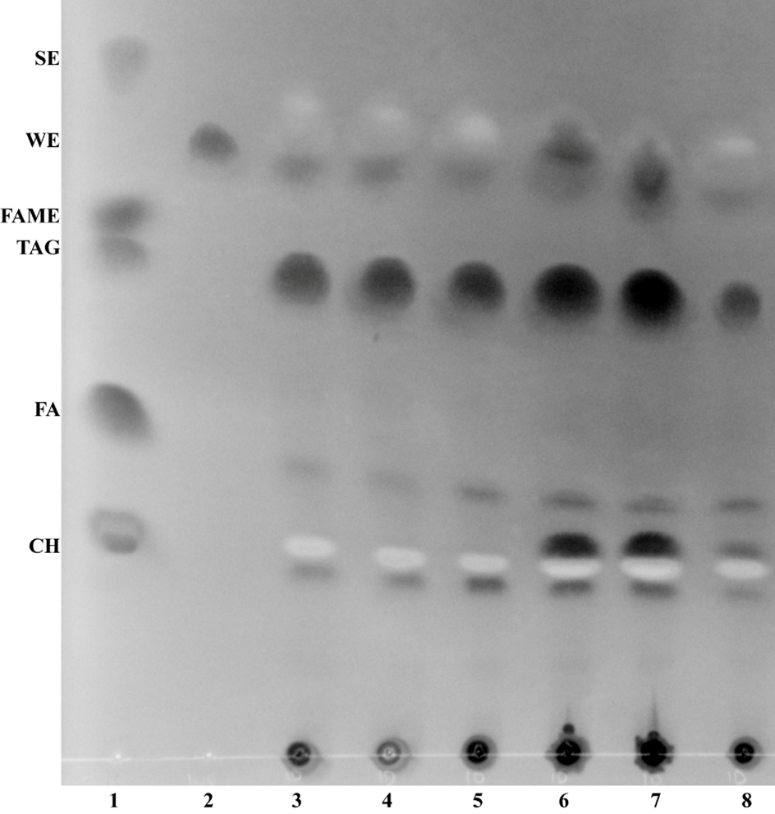


**FOH**

**Figure S4.** Thin layer chromatography. 1, TLC standard = 100 µg cholesterol, 100 µg oleic acid, 100 µg triolein, 100 µg methyl oleate, 100 µg cholesteryl oleate; 2, wax ester standard = 50 µg lauryl laurate (C24:0); 3, strain LW21 clone1; 4, strain LW21 clone 2; 5, strain LW21 clone 3; 6, strain LW23 clone 1; 7, strain LW23 clone 2; 8, strain LW23 clone 3. The TLC was performed as described in Materials and Methods.


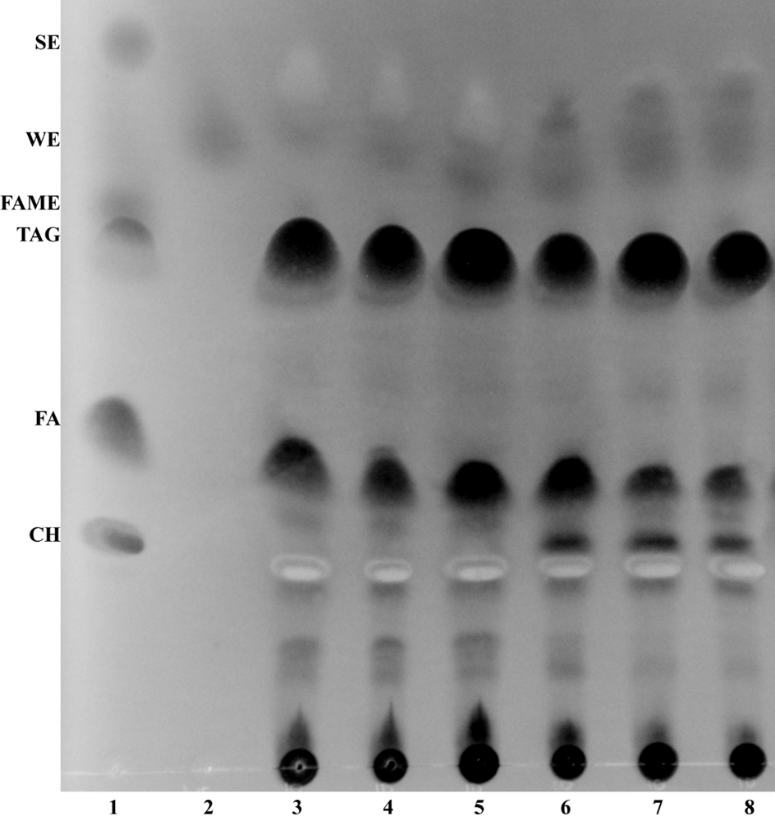


**FOH**

**Figure S5.** Thin layer chromatography. 1, TLC standard = 100 µg cholesterol, 100 µg oleic acid, 100 µg triolein, 100 µg methyl oleate, 100 µg cholesteryl oleate; 2, wax ester standard = 50 µg lauryl laurate (C24:0); 3, strain LW22 clone 1; 4, strain LW22 clone 2; 5, strain LW22 clone 3; 6, strain LW24 clone 1; 7, strain LW24 clone 2; 8, strain LW24 clone 3. The TLC was performed as described in Materials and Methods.


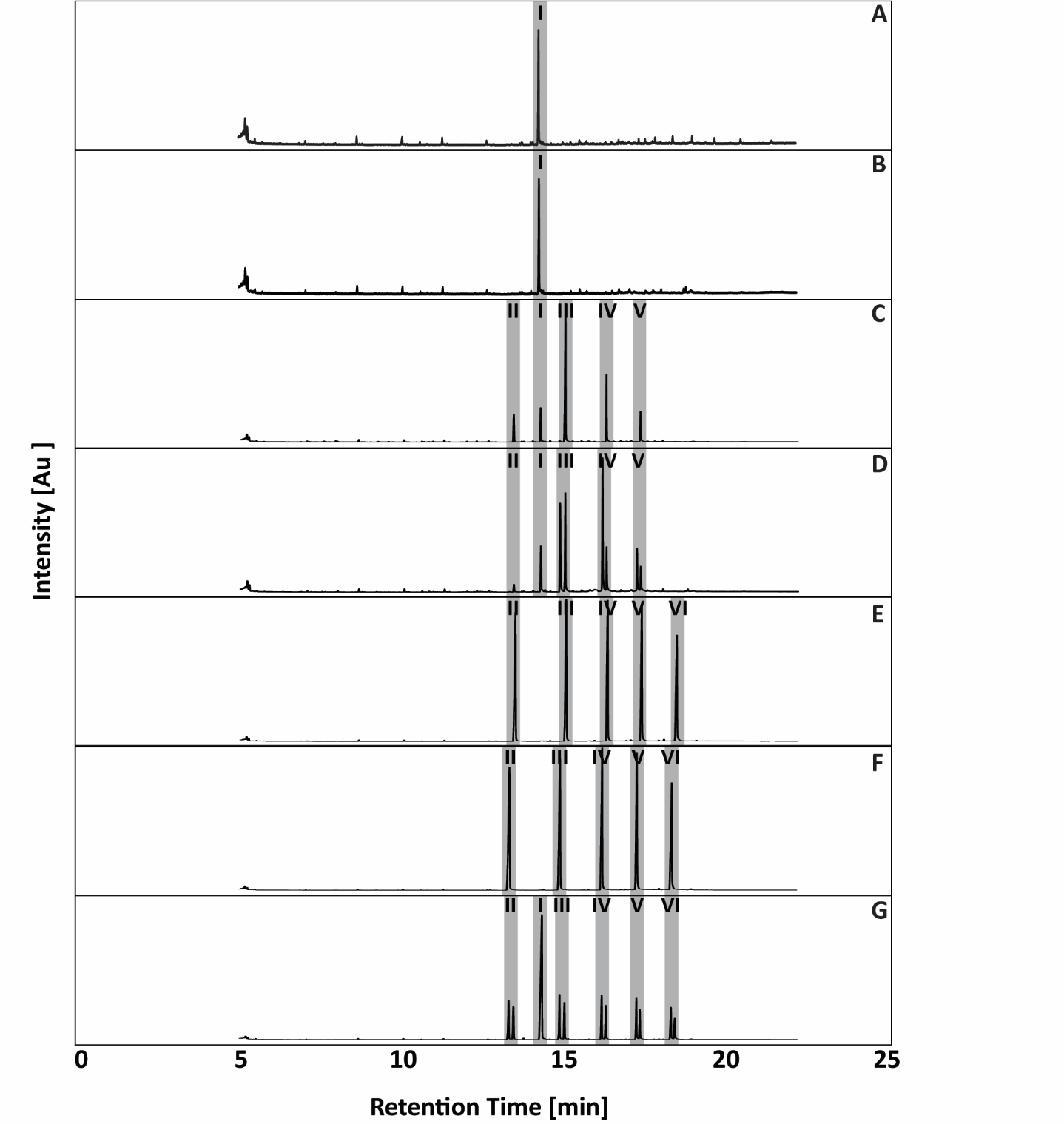
**Figure S6.** GC-FID chromatograms of fatty alcohols (FOHs) isolated from strains LW21 (A), LW22 (B), LW23 (C) and LW24 (D). The peak labeled with (I) corresponds to the internal standard used (C17:0 FOH). A mixture of standards (c = 200 µg/mL) containing the saturated FOHs (II), C16:0; (III), C18:0; (IV), C20:0; (V), C22:0 and (VI), C24:0 is shown in (E). A mixture of standards (c = 200 µg/mL) containing the monounsaturated FOHs (II), C16:1; (III), C18:1; (IV), C20:1; (V), C22:1 and (VI), C24:1 is shown in (F). The chromatogram in (G) shows a combination of the standards (E), (c = 50 µg/mL); (F), (c = 50 µg/mL) and C17:0 (c = 250 µg/mL). The strains were grown for 48 h in minimal medium containing 20 g/L glucose.


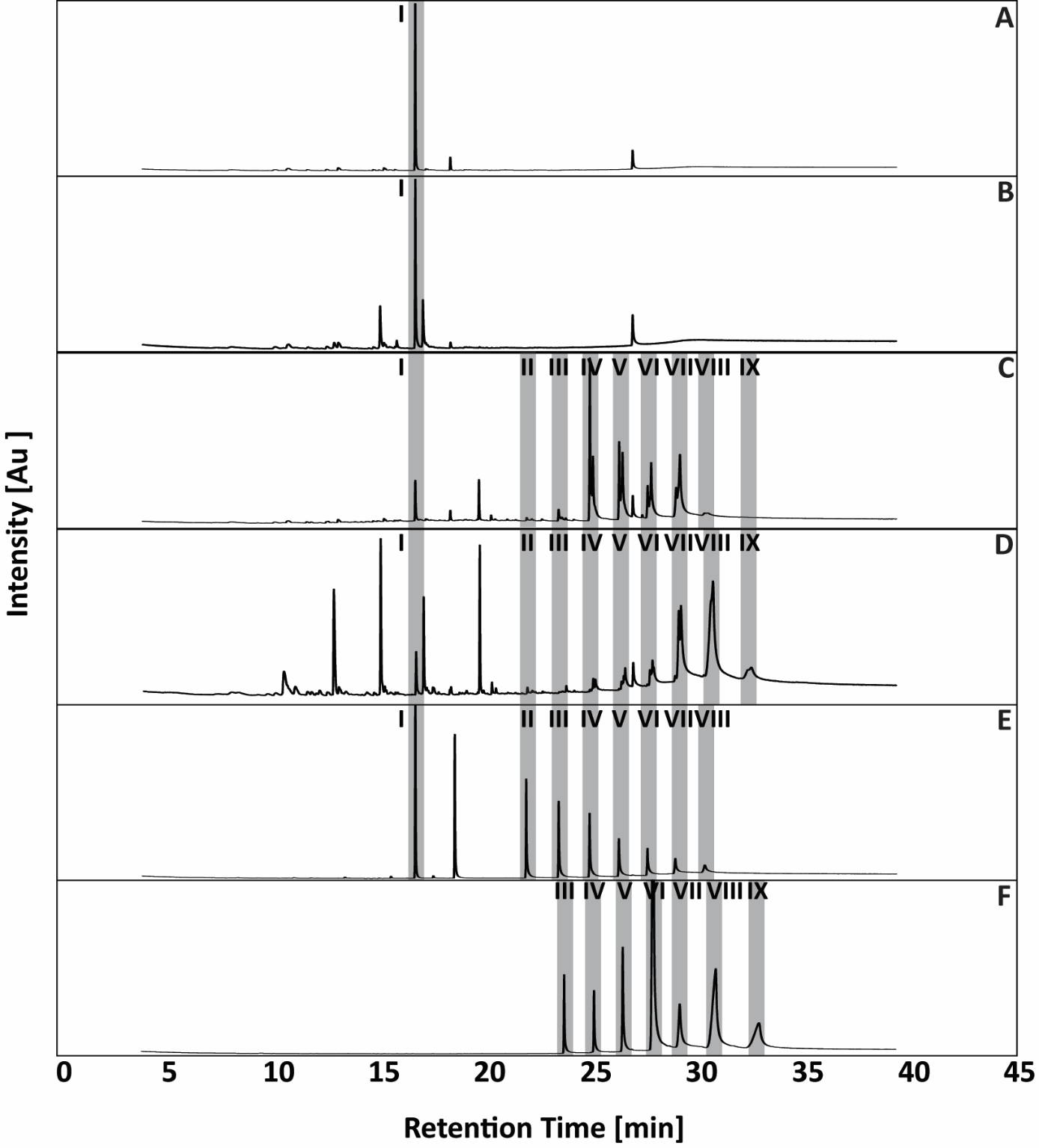


**Figure S7.** GC/MS chromatograms of wax esters (WEs) isolated from strains LW21 (A), LW22 (B), LW23 (C) and LW24 (D). The peak labeled with (I) corresponds to the internal standard used (C24:0 WE). A mixture of standards (c = 10 µg/mL) containing the saturated WEs (I), C24:0; (II), C30:0; (III), C32:0; (IV), C34:0; (V), C36:0, (VI), C38:0; (VII), C40:0 and (VIII), C42:0 is shown in (E). A mixture of standards (c = 10 µg/mL) containing the diunsaturated WEs (III), C32:2; (IV), C34:2; (V), C36:2; (VI), C38:2; (VII), C40:2; (VIII), C42:2 and (IX), C44:2 is shown in (F). The strains were grown for 48 h in minimal medium containing 20 g/L glucose.


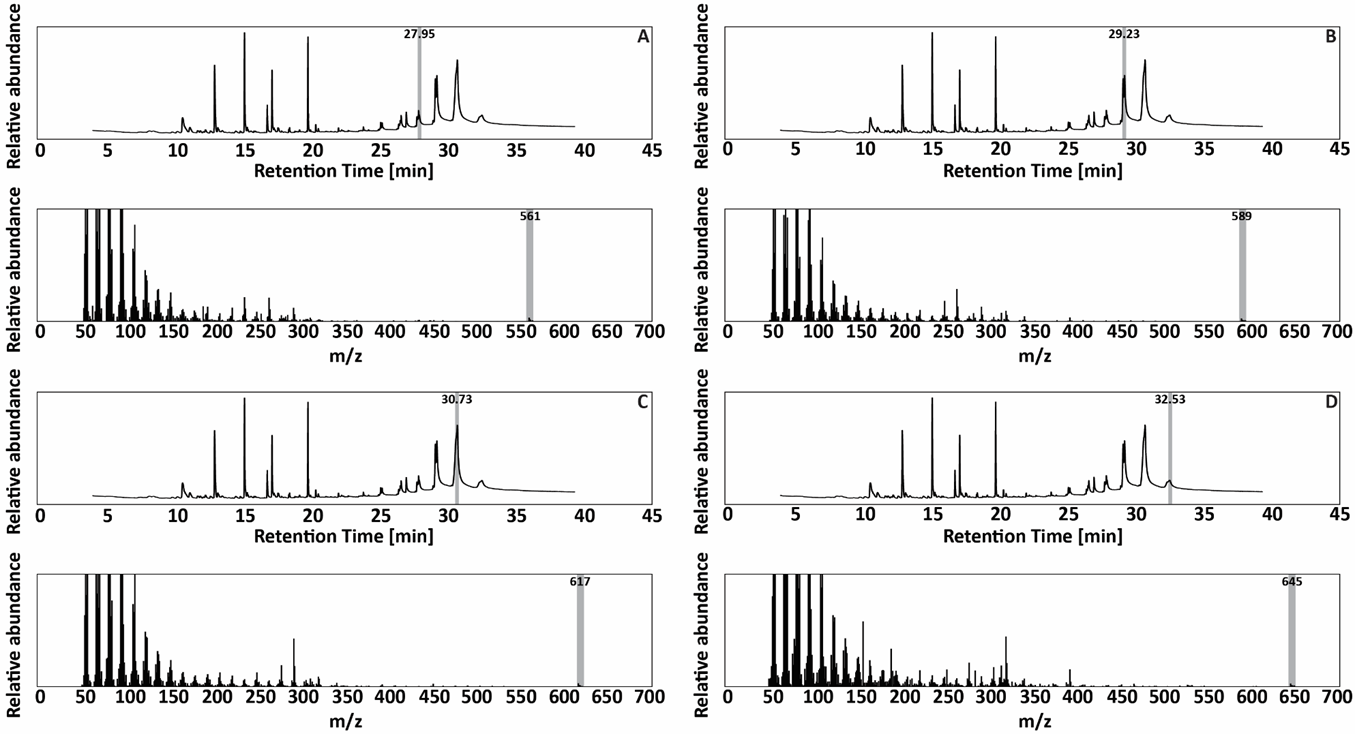
**Figure S8.** Mass spectra of selected peaks of total ion chromatograms of lipids extracted from strain LW24. Mass spectrum of the C38:2 wax esters (WEs) (MW = 561 g/mol), eluting after 27.95 min with the specific m/z peak of 561 (A). Mass spectrum of the C40:2 WEs (MW = 589 g/mol), eluting after 29.23 min with the specific m/z peak of 589 (B). Mass spectrum of the C42:2 WEs (MW = 617 g/mol), eluting after 30.73 min with the specific m/z peak of 617 (C). Mass spectrum of the C44:2 WEs (MW = 645 g/mol), eluting after 32.53 min with the specific m/z peak of 645 (D).
